# Meaning of nutrition for cancer survivors: a photovoice study

**DOI:** 10.1136/bmjnph-2023-000822

**Published:** 2024-02-21

**Authors:** Niamh O’Callaghan, Pauline Douglas, Laura Keaver

**Affiliations:** 1 Department of Health and Nutritional Science, Atlantic Technological University, Sligo, Ireland; 2 Health and Biomedical Strategic Research Centre (HEAL), Atlantic Technological University (ATU), Sligo, Ireland; 3 Ulster University, Coleraine, UK; 4 NNEdPro Global Institute for Food Nutrition and Health, Cambridge, UK

**Keywords:** Nutrition assessment

## Abstract

**Background:**

Little is known about how cancer survivors perceive nutrition through the cancer experience and how those perceptions may influence their diet.

**Aims:**

This study aimed to capture the meaning of nutrition for cancer survivors who are post-cancer treatment using a participatory photography method known as photovoice.

**Methods:**

Wang and Burris’s photovoice procedure was followed. Recruitment took place via email through existing links with participants from a previous quantitative study. The participants were tasked with taking photographs to represent the meaning of nutrition for them post-treatment. Group workshops and semistructured interviews were conducted to facilitate reflection, dialogue and analysis. Data analysis followed Braun and Clarke’s six-phase thematic analysis.

**Results:**

One man and seven women (n=8) across the Island of Ireland were recruited. Participants identified six themes (illustrated with photographs): (1) Fresh is Best, (2) Be kind to yourself, (3) Building Blocks. Be Informed., (4) Post-Treatment Healing Changes, (5) Chemo Rituals and (6) Food for the Soul–Healthy Mind. Healthy Body.

**Conclusions:**

Participants displayed a holistic approach to a healthy lifestyle for recovery post-treatment and maintaining health. While diverse, participants made post-treatment nutritional changes by introducing and eliminating certain foods or food groups. All agreed that being informed and building nutrition knowledge are essential. It is important to clarify the implications cancer has had on diet and health when providing nutrition guidance to ensure that it is appropriate and specific.

**WHAT IS ALREADY KNOWN ON THIS TOPIC**

- Cancer survivors recognise the importance of nutrition and are motivated to seek information about dietary changes to improve their long-term nutritional and health outcomes.

**WHAT THIS STUDY ADDS**

- Our participant-generated findings are of practical use in illustrating the perspectives on diet and health in this population.

**HOW THIS STUDY MIGHT AFFECT RESEARCH, PRACTICE OR POLICY**

- This study emphasises how crucial it is to consider and clarify cancer’s implications post-treatment when providing nutrition guidance and advice to ensure that it is appropriate and specific.

## Introduction

The number of cancer survivors has increased by more than 50% in the last decade, and currently, more than 200 000 individuals in Ireland live with and beyond cancer.^1^ A cancer diagnosis can catalyse change in many health behaviours, including diet.^2^ Despite some studies showing improved nutritional quality and adherence to World Cancer Research Fund (WCRF) recommendations in cancer survivors,^3^ other studies have shown lower diet quality, poor adherence to dietary guidelines and unwanted weight gain compared with the general population.^4–6^

Nutrition can be an overlooked and under-resourced part of the cancer care continuum, particularly in Ireland. Only 39% of the cancer survivors in a recent nationwide study by Sullivan *et al* 7 were referred to a registered dietitian, despite 89% of the cohort rating nutrition as essential. Furthermore, over half of the participants (57%) wanted clarification regarding the frequently contradictory nutrition advice presented by others and in the media.

It is important to take into account the effects of cancer on dietary intake as well as the importance of nutrition for cancer survivors post-treatment when providing post-diagnosis nutritional support. Thus, this study aims to use a visual methodology called photovoice to understand the significance of nutrition for Irish cancer survivors.

To better understand health concerns from the perspectives of individuals who are experiencing them, to motivate intervention and clarify health issues, public health researchers have increasingly started using visual methodologies.^8^ Photovoice’s four main pillars—images, group conversation, participant growth and action—can be customised to fit the demands of any project and target audience. The ultimate objectives of photovoice have been focused on comprehending health action and advocacy, individual and community empowerment, and health intervention across a variety of communities.^8–10^ Participatory research techniques can improve our understanding of nutrition in cancer survival by allowing participants to actively contribute to knowledge creation rather than acting as passive subjects.^11^

## Materials and methods

### Study design

The researchers sought to address the question, ‘What is the meaning of nutrition for Irish cancer survivors?’ using a participatory descriptive qualitative study. Accordingly, the study objective was loosely structured so the participants could take photos that they felt reflected their relationship with nutrition and how it impacted their life. Participants were encouraged to interpret the photography task in whatever way made the most sense for them.^12^ The study team did not wish to narrow the photography task as they wanted to obtain the best insight into the participants’ relationship with their dietary choices and nutrition behaviours.

This study was conducted by an academic research team collaboratively with the study participants, using guidance as outlined in Wang and Burris’s field.^13^ NO’C completed photovoice’s comprehensive online training course (https://photovoice.org) and facilitated the group workshops and semistructured interviews. The Consolidated Criteria for Reporting Qualitative Research checklist is reported in the online supplemental material 1.

10.1136/bmjnph-2023-000822.supp1 Supplementary data

### Inclusion criteria and recruitment

Inclusion criteria included cancer survivors (aged ≥18 years) who had completed active cancer treatment (≥6 months) and were living across Ireland. Participants had to own a smartphone. Photovoice studies often have small sample numbers since their main goal is to completely understand participants’ perspectives and encourage them to freely communicate their experiences and thoughts. For recruitment, the study was circulated on social media and throughout cancer networks across Ireland. Of the eight participants who responded, five responded from a more extensive quantitative study on the nutrition practices of cancer survivors in Ireland,^14^ while three responded to online recruitment calls such as e-newsletters from associated patient groups or social media platforms.

### Photovoice procedure

The photovoice project was conducted in four distinct phases, occurring sequentially over 8 weeks in autumn 2021, and conducted online via Microsoft Teams.

#### Introductory group workshop

Introductory 1-hour workshops facilitated the participants’ preferred time schedules. To accommodate everyone’s schedule, a total of four workshops were held; therefore, some participants completed the introduction workshop in a group setting, while others did it individually. In each, the facilitator explained ground rules, defined participatory photography and gave an overview of the project. Exercises in visual literacy and photo analysis were also conducted. Photovoice’s ‘four fs’ (frame, focus, follow through and flash) to taking better photographs were demonstrated. Potential ethical issues were discussed, such as identifying appropriate and inappropriate pictures (eg, illegal, unsafe) and how to obtain consent to photograph another person. Participants were then given a photography assignment titled ‘The meaning of nutrition for Irish cancer survivors’ and asked to take a minimum of five shots each using their smartphone during the following 2 weeks. There was no maximum number of pictures. The participants were able to express any concerns on ethics or the project task during the workshop.

#### Taking the photographs

Participants were given 2 weeks to take photographs. The cohort submitted 52 photographic images in total (ranging from 4 to 14 photographs each).

#### Individual interviews

Individual semistructured interviews were conducted at a time convenient for each participant. During the interview, participants were asked to share all photographs that they captured. This interview provided an opportunity with the facilitator to caption their selected pictures for the final workshop 2 weeks later. Where required, a commonly deployed method, the ‘SHOWED’ mnemonic, was used to discuss and caption each photograph. This includes six questions for each photograph: (1) what do you See here?; (2) what is Happening here?; (3) how does this relate to Our lives?; (4) Why does this situation, concern or strength exist?; (5) how can we be Empowered by this?; (6) what can we Do about it? If participants required more time to create captions for their pictures, they had until the final group workshop. The duration of the interviews ranged from 30 to 60 minutes.

#### Final group workshop

To start the final group workshop, the facilitator introduced ground rules. Each participant introduced themselves to the group, discussed their cancer diagnosis and previous treatments received and explained their current perspective on nutrition. Each participant then presented their photos and captions to the broader group; this took approximately 20 minutes for each participant. A mini-exhibition of all photographs was then assembled using an unsystematic wall layout on a virtual platform called Padlet (http://padlet.com). The group had an opportunity to discuss the pictures collectively and then clustered the photographs into similar groupings using colour coding. The participants were asked to discuss and identify potential themes represented by these clusters; some photographs were regrouped until the cohort had agreed on the selected photographs. The participants and facilitator discussed theme names by discussing the similarities and differences within the photographs in each theme. Shortlisted images and captions were circulated to all participants by email post-workshop to ensure they had been accurately captured. All photographs and captions can be seen in the online supplemental material 2 of this paper. The final workshop was audio-recorded and transcribed for reliability.

10.1136/bmjnph-2023-000822.supp2 Supplementary data

### Data analysis

In the final group workshop, participants acted as co-researchers to inductively develop six analytical themes representing the meaning of nutrition for them. These were then deductively applied by the facilitator to the rest of the data (eg, interviews and photos). This is a grounded practice known as ‘live coding’, aiming to maximise the researcher’s interaction with the data and ensure analytical rigour.^15^ It involves manual coding while simultaneously listening to the audio-recording of the workshop. Braun and Clarke’s six-step thematic analysis was conducted to determine if any additional themes arose during the discussions and Microsoft Excel was used for data organisation and mapping.^15^ No additional themes were found in this process.

## Results

### Study participants

Eight cancer survivors were recruited (seven women (breast cancer) and one man (prostate cancer)). Age ranged from 45 to 59 years old (mean age: 51 years). The majority (n=7) had completed treatment in the last 5 years. All participants had higher education (third level) of varying levels and half were in full-time employment.

### Visual and narrative themes

Participants generated six main themes from the pictures: (1) Fresh is Best, (2) Be kind to yourself, (3) Building Blocks. Be Informed., (4) Post-Treatment Healing Changes, (5) Chemo Rituals and (6) Food for the Soul–Healthy Mind. Healthy Body.

#### Theme 1: Fresh is Best

**Figure 1:**
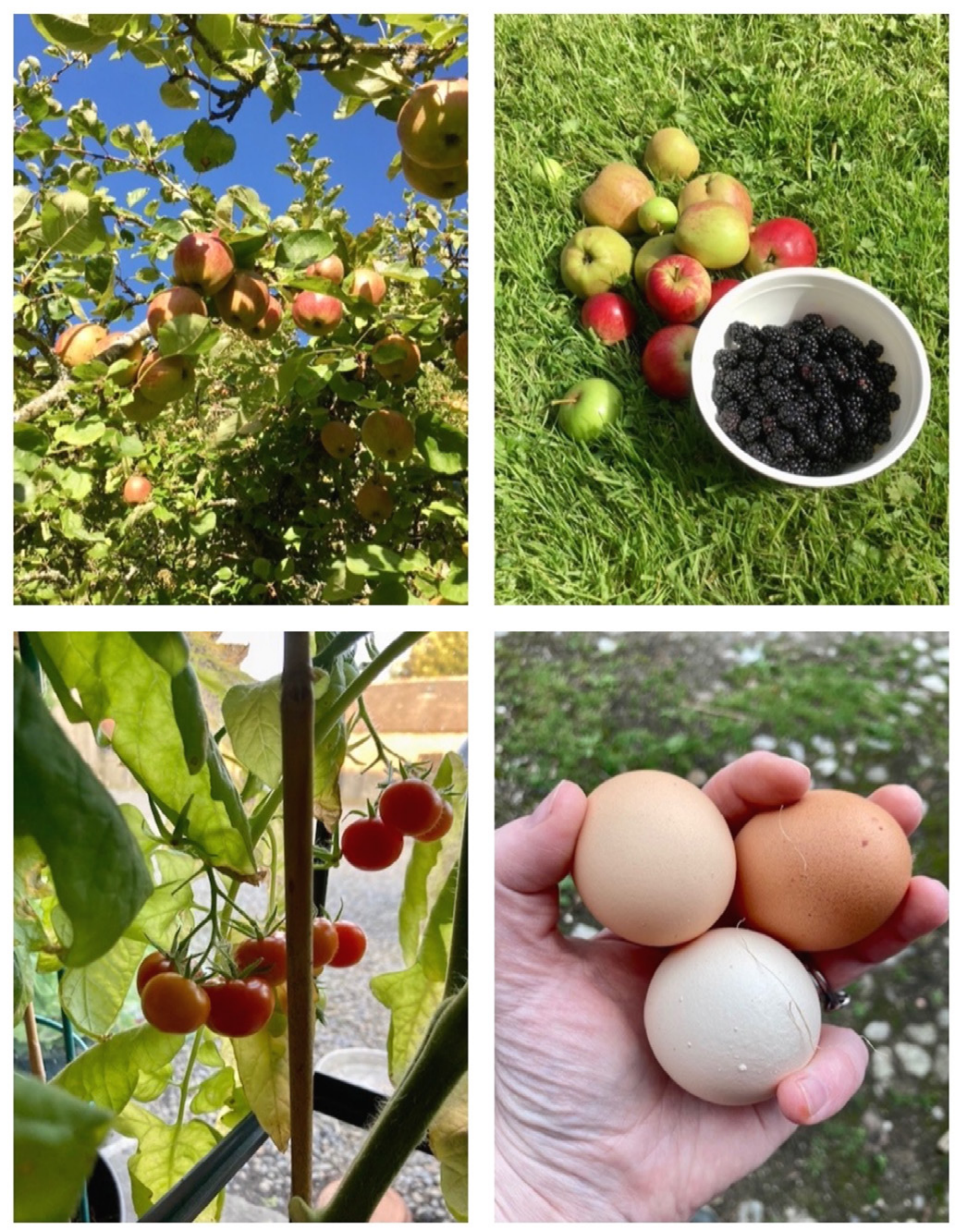
Living the good life! I had all my treatment in Dublin, and we moved home a year after I finished treatment. I had always loved to forage, and now I am also growing my vegetables, and we have our hens!

Participants highlighted the importance of fresh fruits and vegetables for recovery and maintaining health, with an emphasis on freshness and quality. Some only sourced locally grown produce; others purchased only organic produce, while one cancer survivor home foraged and is undertaking ‘an organic course at the moment’ to change a family farm’s status to organic (figure 1). A participant described a local greengrocer as a ‘little oasis’ and explained many benefits of sourcing local: ‘the selection of fruit and vegetables is always so fresh and full of colour’. One cancer survivor mentioned nutrition post-treatment involved going ‘back to basics’, explaining, ‘I went back doing what our parents ate, everything fresh and out of the ground—no added preservatives. We eat as natural as we can, and the fewer processes that food goes through, the better it is for you.’ One participant talked about the struggle in ‘trying to follow an organic plant-based diet’.

#### Theme 2: Building Blocks. Be Informed.

**Figure 2:**
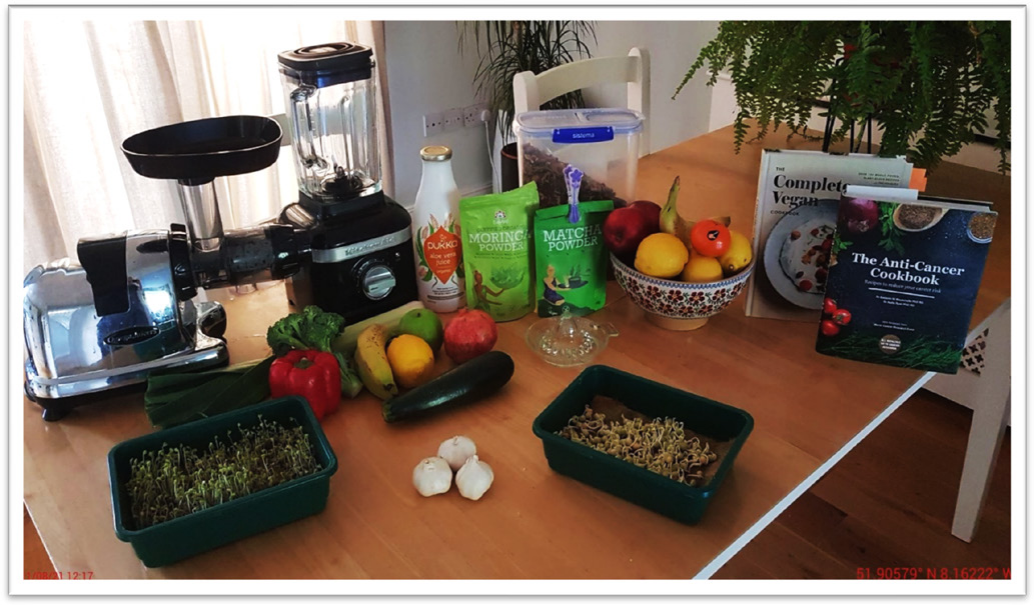
New diet and nutrition.

The participants shared the practice of self-directed research through various methods, including online, through books and local food producers. Different dietary choices and beliefs were present for the cohort; however, all agreed it is essential to be informed and build on nutrition knowledge. Some participants explicitly viewed cancer as a teachable moment for behaviour change. In figure 2, a participant gives a ‘snapshot’ of a new diet post-cancer treatment. This participant undertook their own research and rationalised several dietary changes: ‘I took from what I’m reading, I’m trying to follow an organic plant-based diet, so in terms of white sugar, white flours, all those things are all out the window because they’re not healthy, processed meats, I steer away from them, you know, dairy is well’. Moreover, another cancer survivor presented an image of a local award-winning butcher and expressed how informed she feels on the source of the product: ‘these guys have won so many awards for their meats, they grow their meat for want of a better word, you know the meat is good. You know there are very few chemicals in it.’ While the group views on dietary choices differed, the cohort did note the importance of being adequately informed.

#### Theme 3: Be kind to yourself

**Figure 3:**
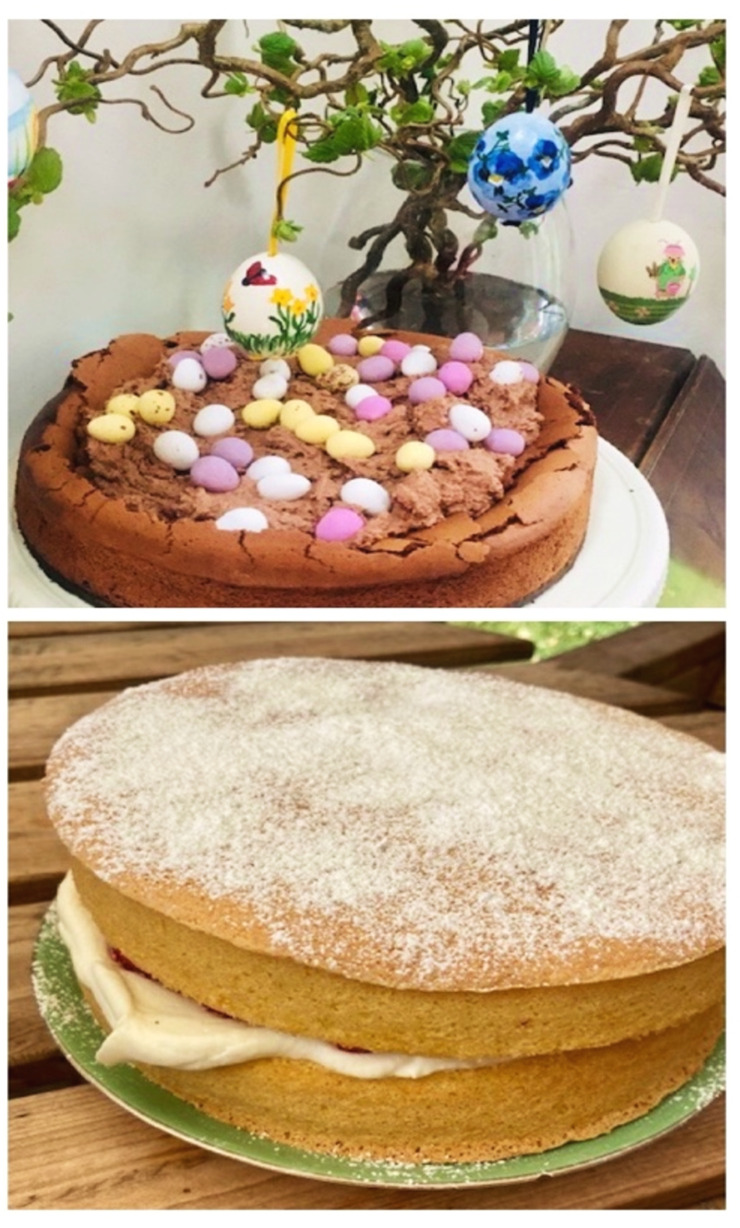
We celebrate every milestone and festival with cake!

Broadly, this theme captured forms of celebration, as reflected in figure 3, and an approach to diet as ‘everything in moderation’. One participant outlined the importance of celebration: ‘now we celebrate all the milestones; I was 41 when I was diagnosed. I had three small children, now we celebrate Easter, we celebrate Christmas, we celebrate anything and everything.’ Food as a symbolic meaning for family time and social connections was also classified into this category. One individual shared an image containing ingredients for a family roast dinner highlighting: ‘an old-fashioned Sunday roast, good food and good company’.

#### Theme 4: Post-Treatment Healing Changes

**Figure 4:**
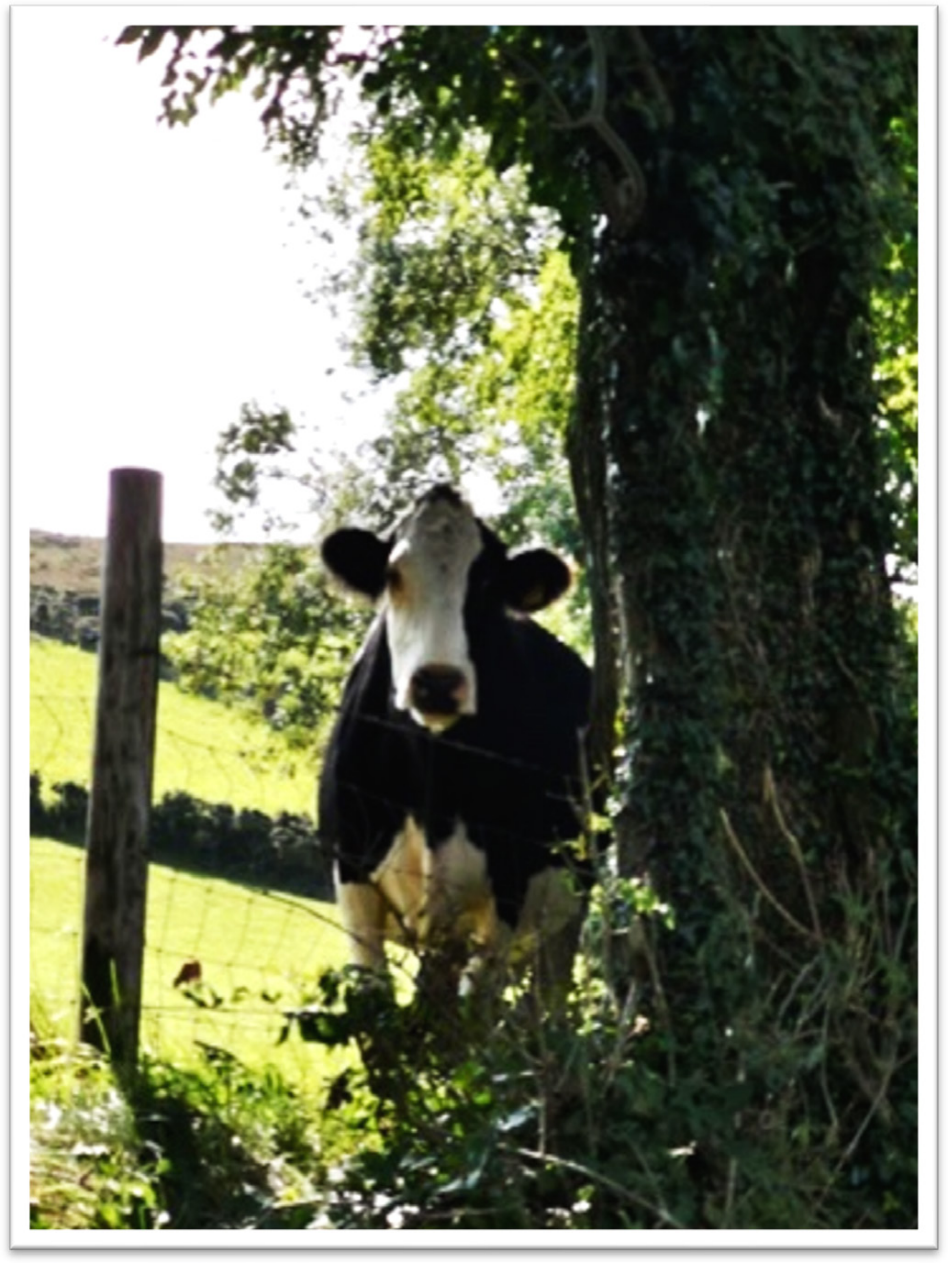
This is the only dairy I see.

Participants made post-treatment changes by introducing and eliminating certain foods or food groups. While diverse, all the cohort agreed these fit the one theme, ‘Post-Treatment Healing Changes’, where the collective objective of these changes aimed to ‘heal from within’. One participant explained an intake of vitamin and mineral supplements which she described as part of a ‘post-treatment routine’. A common addition and photograph presented among the participants was an intake of foods with believed anti-inflammatory properties, such as mushrooms, garlic and turmeric. One cancer survivor described how ‘turmeric is something now that I have started putting into almost everything I’m cooking because I have neuropathy, and I just feel I’m very inflamed in general, I find I don’t know whether it’s working or if it’s in my head, but anyways, turmeric goes into everything’. Figure 4 relates to one participant cutting dairy out of her diet initially post-treatment and now only occasionally eats dairy. This theme may seem to overlap with theme 2, ‘Building Blocks. Be Informed’. However, the participants felt it was important to present them separately. The photographs in theme 2 were more informed choices than in this theme, where the participants’ preferences were individually based.

#### Theme 5: Chemo Rituals

**Figure 5:**
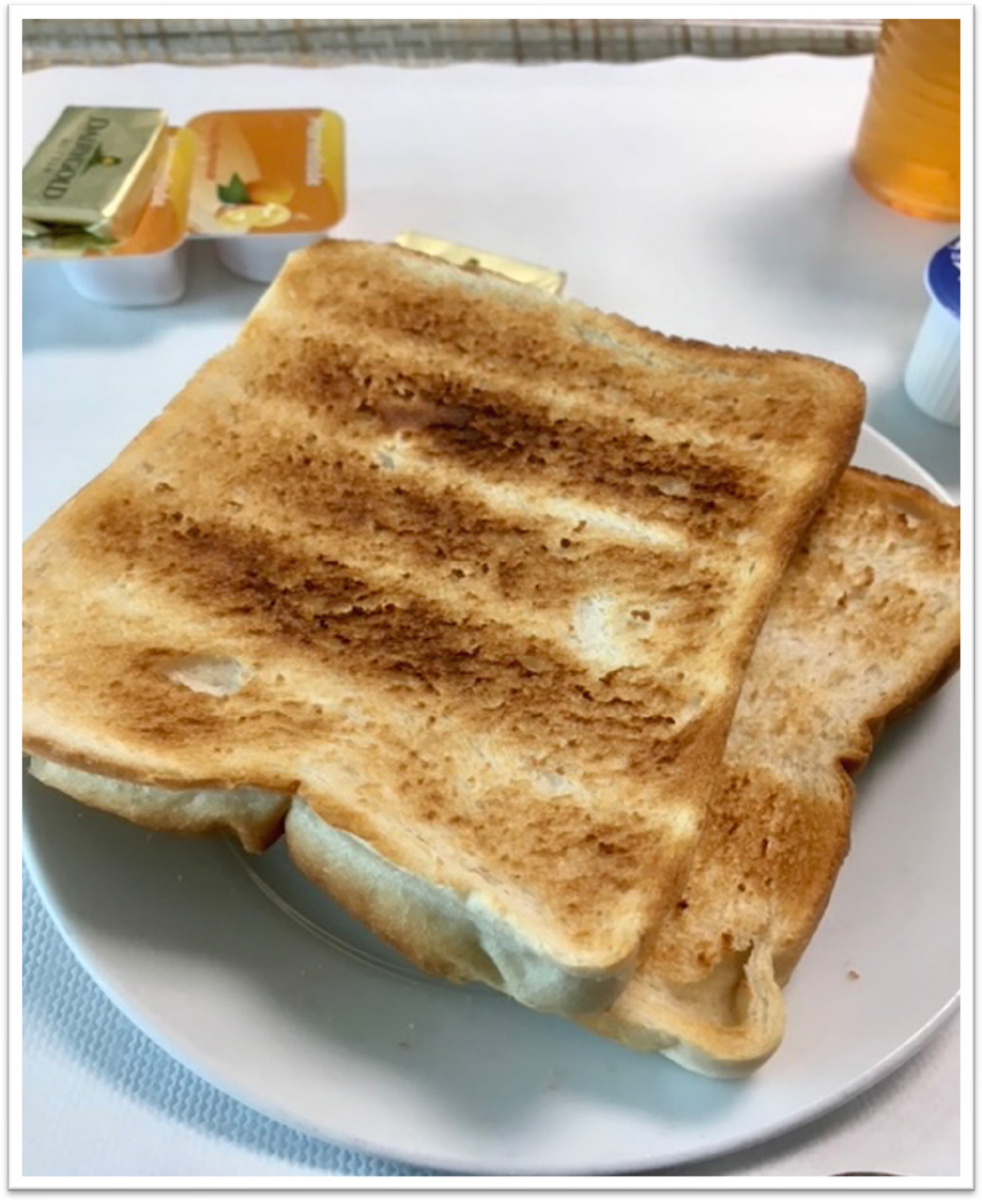
Chemo breakfast.

Among the cohort, certain foods and dietary practices were associated with receiving chemotherapy treatment. One participant presented an image of coconut water where she shared her chemotherapy experience: ‘every session before chemo, I was drinking litres of coconut water, research in the UK has shown your blood count will never be wrong for chemotherapy’. Figure 5 presents a breakfast of white toast, which participants agreed will always be identified as a ‘chemotherapy breakfast’.

#### Theme 6: Food for the Soul–Healthy Mind. Healthy Body.

**Figure 6:**
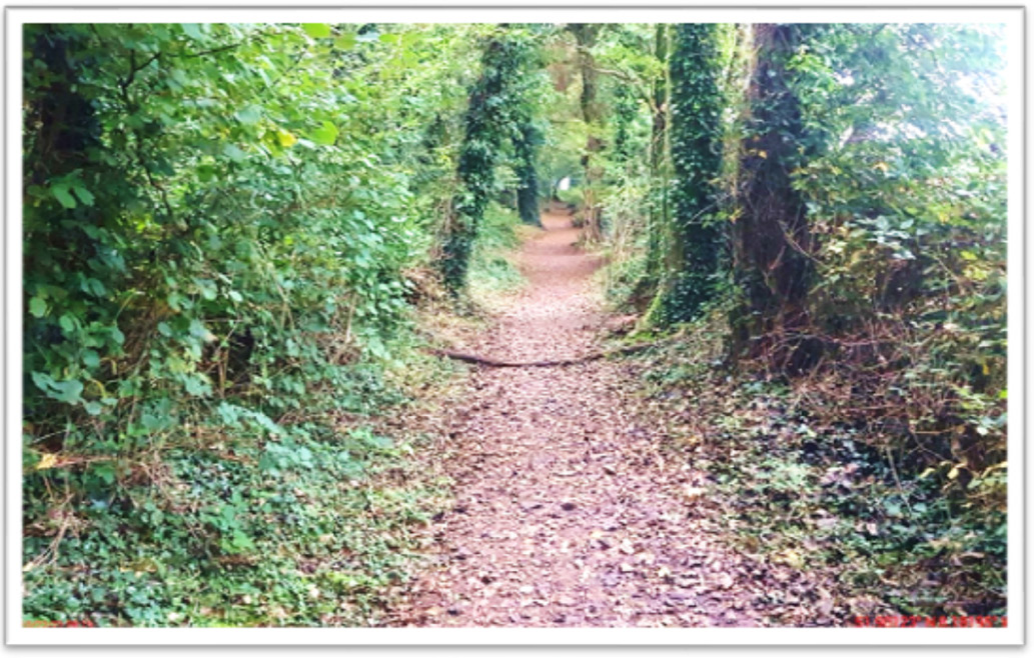
This here is de-stress, but it’s more, as you see, the very middle centre, that’s the light at the end of the tunnel in a cancer journey and the broken branch across the photo signifies the barriers you may encounter, but there is always light at the end of the tunnel.

When describing the theme, Food for the Soul–Healthy Mind. Healthy Body., participants remarked on regaining energy and described outside movement and exercise as ‘food for the soul’. The term ‘exercise’ was associated with life pre-cancer, with one individual now opting to use the word ‘movement’: ‘I hate the word exercise because that’s what I did pre-cancer but post-cancer, its movement and anything that you can do out in the fresh air’. Although the images within this theme embodied outside environments, including photographs of mountains and the seaside, the participants associated it with their overall nutrition and health. One participant explains: ‘These things are nutrition in their way as well, nutrition for the soul, it’s not just the food, I mean the food is important, but it can’t be in isolation, you know?’. Images within this theme had ambiguous meanings, including symbols of positivity and hope. For example, one embodiment portrayed a ray of light from the sun shining through trees, and ‘was my example of a symbol of hope’ (figure 6).

## Discussion

Our project sought to capture the meaning of nutrition for Irish cancer survivors using a method of participatory photography known as photovoice. Emphasis was placed on dietary intake of fresh fruits and vegetables to recover and maintain health. Participants expressed celebration through food with a reminder to ‘be kind to yourself’. Different dietary choices and beliefs were present. However, the cohort agreed it is essential to be correctly informed and build on nutrition knowledge. While diverse, participants made post-treatment ‘healing’ changes to their dietary intake by the process of introducing and eliminating certain foods or food groups. Additionally, the theme ‘chemo rituals’ was included as particular foods are associated with their time spent receiving chemotherapy. Although the focus was on ‘nutrition’, all participants had photographs embodying the natural landscapes or exercise/movement, describing it as ‘food for the soul’.

The group perspectives on the origin of products are very similar to a study by Chapman and Beagan^16^ on women’s beliefs about diet and breast cancer. In this particular study, the authors defined a ‘traditional’ or ‘old-fashioned’ perspective of non-processed meats and vegetables for people with a European cultural heritage. It was distinct from the other perspectives in that it was the one that valued the consumption of meat. It also placed less emphasis on health concerns, defining ‘good food’ or ‘good eating habits’ based on tradition and enjoyment as much as health issues. In this study, another perspective was classified as an ‘alternative’ perspective which focused on the role of toxins, carcinogens and protective factors in food in affecting cancer risk. The traditional and alternative outlooks support the perspectives of our cohort, and some similar views occur. Still, others offer opposing opinions, such as the importance of locally sourced meat (traditional perspective) and eating only organic plant-based food (alternative perspective).

A cancer diagnosis led to dietary changes in a cohort, with some avoiding dairy products due to carcinogenic properties, while others added turmeric, garlic and mushrooms for anti-inflammatory benefits, similar to Irish cancer survivors’ advice on avoiding certain foods.^17^ Although evidence indicates the role of inflammation in cancer risk,^18 19^ modifiable lifestyle factors associated with inflammation, such as diet quality,^20^ are understudied and relatively sparse. Furthermore, the WCRF/American Institute for Cancer Research (AICR) guidelines for cancer survivors for cancer prevention focus on overall dietary patterns rather than intake levels of individual nutrients and compounds.^3^

The use of vitamin and mineral dietary supplements among cancer survivors is commonly reported, with prevalence rates in breast, prostate and colorectal cancer survivors ranging from 50% to 85%,^21 22^ with 15–30% of cancer survivors initiating supplement use after their cancer diagnosis.^23^ Similarly, the intake of dietary supplements was described as a ‘post-treatment routine’ for some participants in the present study. In general, no obvious reasons for taking them were given, and the goal seemed to be health improvement rather than particular anticancer properties. Conversely, the WCRF/AICR strongly encourages cancer survivors to obtain their nutritional needs through a healthy balanced diet instead of taking supplements,^3^ as the benefit of such supplements is questionable.

Our cohort reported not receiving any nutritional support or dietary information from a health professional. They obtained their knowledge through self-directed research online and from various books. The use of informal sources such as ‘online’ may partially explain why some of our participants’ perspectives on dietary modifications were not in line with current evidence (eg, high intake of anti-inflammatory foods and nutritional supplements being beneficial).^8^

In the theme ‘Chemo Rituals’, it was clear from the images that the associations with, for example, white toast, energy snack balls and coconut water, continue post-treatment. It should be noted how these associations could impact dietary intake or certain practices. When providing nutrition guidance, it is essential to consider and clarify all implications cancer has had.

The participants remarked on regaining energy and described outside movement and exercise as ‘food for the soul’. Engaging in physical activity and training has many beneficial effects on health-related quality of life domains in cancer survivors, including fear of recurrence (eg, breast cancer), emotional well-being, sexuality, sleep disturbance, social functioning, fatigue and pain.^24 25^ The benefits of physical activity are further reinforced by results of observational studies reporting that regular physical activity of 3–5 days a week for a minimum of 30 min per activity is correlated with a reduction in mortality and all-cause mortality in early-stage breast^26^ and prostate cancer.^27^ Although research shows that nearly two-thirds of cancer survivors do not meet national physical activity guidelines, those who meet the guidelines report better quality of life in multiple domains than less active individuals.^28 29^ The study found that participants in a holistic approach to physical activity, such as rowing and walking, experienced positive mental health outcomes and interpreted natural landscapes as symbols of hope. The participants shared a mutual outlook on creating therapeutic landscapes within nature, which they described as positively impacting emotional and psychological health.^30^

This methodology has the potential to enhance greatly what healthcare professionals can know and understand about the lived experiences of cancer survivors regarding their nutrition practices and dietary intake. It is an illustration of how the photovoice methodology may support collaboration among healthcare professionals and cancer survivors within this topic but also to support other needs of this cohort. The methodology encourages patient and public participation in research, values life experiences, and offers a creative platform to collaborate and advocate.

As with any qualitative study, the present study has certain limitations that should be considered while interpreting the findings. The sample used here was small but standard for a photovoice study and similar to other photovoice projects with cancer survivors and patients.^31^ Notably, the photovoice approach necessitates a consistent commitment from the participants; thus, a smaller sample size is more feasible. Breast cancer survivors are the primary group represented in the study. There was only one male in our study with a different cancer type. However, there were many group agreements throughout the project regardless of gender or cancer type.

Our photovoice project was carried out online due to the COVID-19 pandemic rather than in person face‐to‐face, which is more common for a photovoice project. This may have made the interviews more impersonal, but it had advantages such as less travel and commitment time for the participants. All participants were well educated; future research should seek to explore this topic with cancer survivors of varying education backgrounds. Furthermore, self‐selection bias could have occurred, where those who took part may have a long‐term interest in healthy lifestyles or have become interested since diagnosis.

## Conclusions

The themes presented here are of practical use in illustrating the perspectives of Irish cancer survivors on nutrition. It is essential to consider and clarify the implications cancer has had on diet and health when providing nutrition guidance and advice to ensure that it is appropriate and specific. The photovoice method helped elicit nutrition perspectives of Irish cancer survivors and proved an easy‐to‐use and acceptable process.

## Data Availability

Data are available upon reasonable request.
