# Supplementary material for: Meaning of nutrition for cancer survivors: a photovoice study
Source: BMJ Nutr Prev Health. 2024 Feb 21;7(1):112–8. doi: 10.1136/bmjnph-2023-000822 (PMC11221309; doi:10.1136/bmjnph-2023-000822)
Supplement: Supplementary data [file bmjnph-2023-000822supp001.pdf]

Consolidated criteria for reporting qualitative research (COREQ): a 32-item checklist for interviews and focus groups

Item	Description
Domain 1: Research team and reflexivity	
Personal Characteristics	
1. Interviewer/facilitator	First author (NOC) conducted the photovoice workshops
2. Credentials	NOC: BSc (Hons) Human Nutrition
3. Occupation	NOC: Irish Research Council Government of Ireland Postgraduate Scholar (M.Sc (Science) by Research
4. Gender	NOC: Female
5. Experience and training	NOC: The first author has done previous qualitative projects and attended several trainings and workshops.; Completed Photovoice's comprehensive online training course (https://photovoice.org) and facilitated the group workshops and semi-structured interviews in this study
Relationship with participants	
6. Relationship established	Participants contacted NOC via email to discuss arrangements for the focus groups. Otherwise, participants had no relationship with the research team.
7. Participant knowledge of the interviewer	Participants were informed that the researcher was conducting postgraduate studies in the area of nutrition and that her goal was to understand the meaning of nutrition for cancer survivors living on across Ireland.
8. Interviewer characteristics	The qualitative researcher and both supervisors were closely engaged in the research process and were therefore unable to completely avoid personal bias.
Domain 2: study design	
Theoretical framework	
9. Methodological orientation and Theory	Thematic analysis was used in this study. The participants acted as co-researchers to inductively develop six analytical themes representing the meaning of nutrition for them. These were then deductively applied by the academic researcher to the rest of the data (e.g. interviews and photos). This is a grounded practice known as 'live coding', which aims to maximise the researcher's interaction with the data and ensure analytical rigour, as Maher et al. It involves manual coding while simultaneously listening to the audio recording of the workshop. Braun and Clarke's six-step

Item	Description
	thematic analysis was followed in analysing and Microsoft Excel for data organisation and mapping.
Participant selection	
10. Sampling	This photovoice study was carried out with cancer survivors (aged ≥18 years) who had completed active cancer treatment at least six months previously and were living across Ireland. Participants had to own a smartphone.
11. Method of approach	Photovoice studies often have small sample numbers since their main goal is to completely understand participants' perspectives and encourage them to freely communicate their experiences and thoughts. For recruitment, the study was circulated on social media and throughout cancer networks across Ireland. Of the eight participants who responded, five responded from a more extensive quantitative study on the nutrition practices of cancer survivors in Ireland (19), while three responded to online recruitment calls such as e-newsletters from associated patient groups or social media platforms.
12. Sample size	There were 8 participants in the study, smaller sample size is more feasible for a photovoice project due to the time commitments required from the participants
13. Non-participation	All participants who agreed on a date and time to attend took part in the photovoice study.
Setting	
14. Setting of data collection	Due to the COVID-19 pandemic, all meetings were conducted online via Microsoft Teams.
15. Presence of non-participants	No one else was present besides the participants and researcher.
16. Description of sample	Eight cancer survivors were recruited across the Island of Ireland (seven women (breast cancer) and one man (prostate cancer)). The participants’ age ranged from 45–59 years old at the time of recruitment (mean age of 51 years). The majority (n=7) had completed treatment in the last five years. Half of the cohort were in full-time employment. All participants had higher education (third level) of varying levels.
Data collection	
17. Interview guide	The photovoice project was conducted in four distinct phases, occurring sequentially over two months in the autumn of 2021. The project involved an introductory group workshop, taking the

Item	Description
	photographs, individual semi-structured interviews and one final group workshop, as detailed in the manuscript.<br>During the individual interview, participants were asked to share all the photographs that they captured. Additionally, in these individual interviews, each person had an opportunity with the researcher to caption their selected pictures for the final workshop two weeks later. In a review of the photos, if required, a commonly deployed method called the 'SHOWED' mnemonic was used to discuss each photograph. This mnemonic includes five questions for each photograph: (1) What do you See here? (2) What is Happening here?; (3) How does this relate to Our lives?; (4) Why does this situation, concern, or strength Exist?; (5) How can we be Empowered by this?; (6) What can we Do about it? The 'SHOWED' mnemonic helped each participant in captioning their photographs.<br>The participants were involved in informing and validating findings during the last workshop, while the research team had regular meetings to discuss emerging themes.
18. Repeat interviews	No repeat interviews were carried out.
19. Audio/visual recording	Audio recording was used to collect the data during the final group workshop via Microsoft Teams. The participants were informed the interview was being recorded.
20. Field notes	Field notes were made during and after the group work.
21. Duration	The duration of the individual interviews ranged for each participant, from a minimum duration of 30 minutes to a maximum period of 60 minutes. The group workshop duration was two and a half hours.
22. Data saturation	Several participants had similar photographs and data saturation was discussed amongst the study team.
23. Transcripts returned	Shortlisted images, captions and articles were circulated to all participants by email for feedback and agreement to validate and member-check the pieces and validate results.
Domain 3: analysis and findings	
Data analysis	
24. Number of data coders	The first author and all participants
25. Description of the coding tree	A mini-exhibition of all the photographs was then assembled using an unsystematic wall layout on a virtual platform called Padlet (http://padlet.com). The group had an opportunity to discuss the pictures collectively and then clustered the

Item	Description
	photographs into similar groupings using colour-coding. The participants were asked to discuss and identify potential themes represented by these clusters; some photographs were re-grouped until the cohort had agreed on the selected photos.
26. Derivation of themes	The identified themes are reflective of patterns in the data and aim to provide a unified picture. The participants and researcher discussed theme names by discussing the similarities and differences within the photographs in each theme.
27. Software	Data was managed by (http://padlet.com)
28. Participant checking	Shortlisted images, captions and articles were circulated to all participants by email for feedback and agreement to validate and member-check the pieces and validate results.
Reporting	
29. Quotations presented	Participant quotations and images were presented to illustrate the themes/findings. They were not identified in order to preserve confidentiality so no one person's story could be put together and identified.
30. Data and findings consistent	Yes, there is consistency.
31. Clarity of major themes	All major themes clearly presented in the findings.
32. Clarity of minor themes	N/A
