# Supplementary material for: Meaning of nutrition for cancer survivors: a photovoice study
Source: BMJ Nutr Prev Health. 2024 Feb 21;7(1):112–8. doi: 10.1136/bmjnph-2023-000822 (PMC11221309; doi:10.1136/bmjnph-2023-000822)
Supplement: Supplementary data [file bmjnph-2023-000822supp002.pdf]

# The meaning of nutrition for Irish cancer survivors: A photo voice study

## Theme 1: Fresh is Best

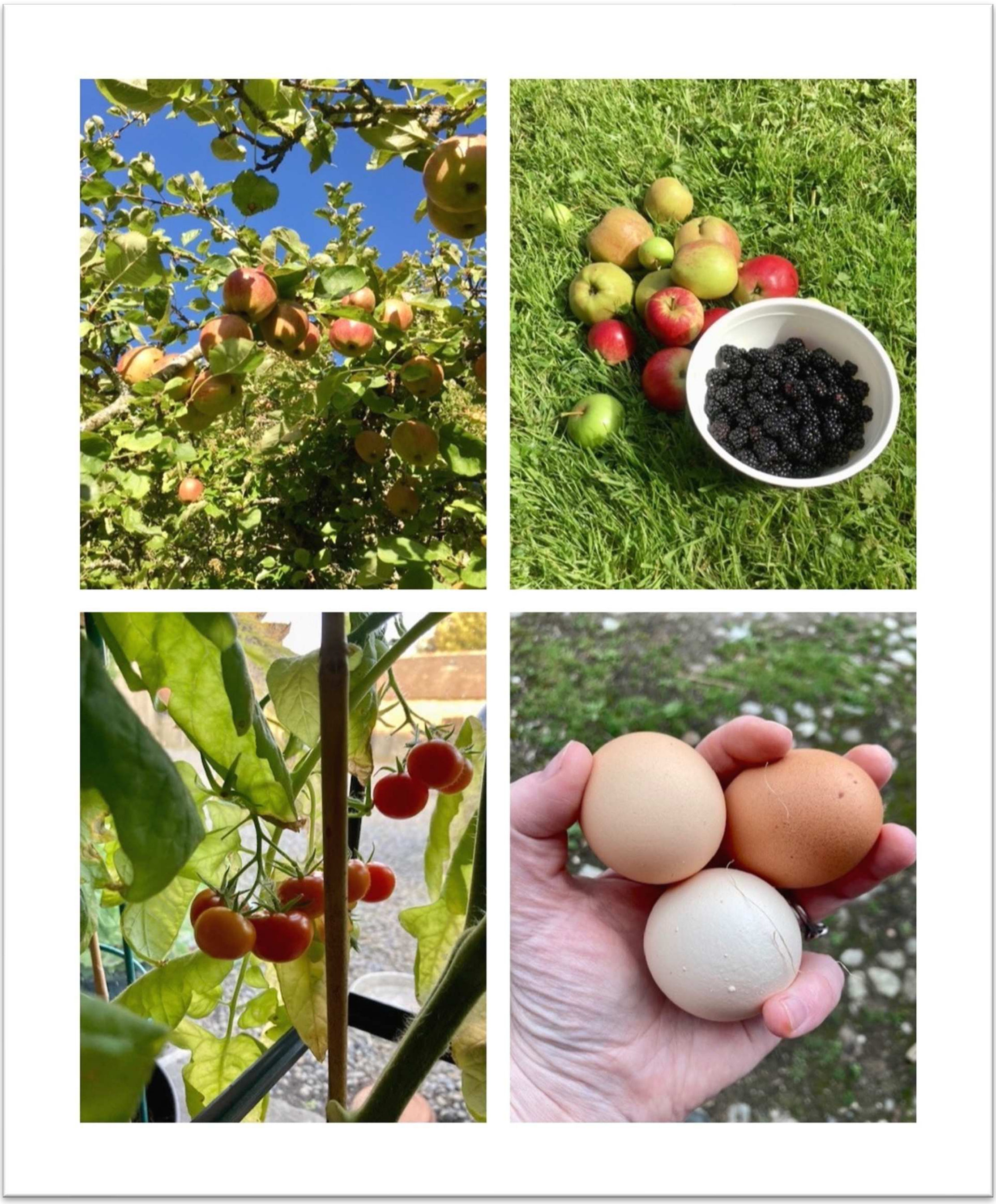

*Figure 1 - Living the good life! I had all my treatment in Dublin and we moved down home a year after I finished treatment. I had always loved to forage and now I am also growing my own vegetables and we have our own hens!*

The meaning of nutrition for Irish cancer survivors: A photo voice study

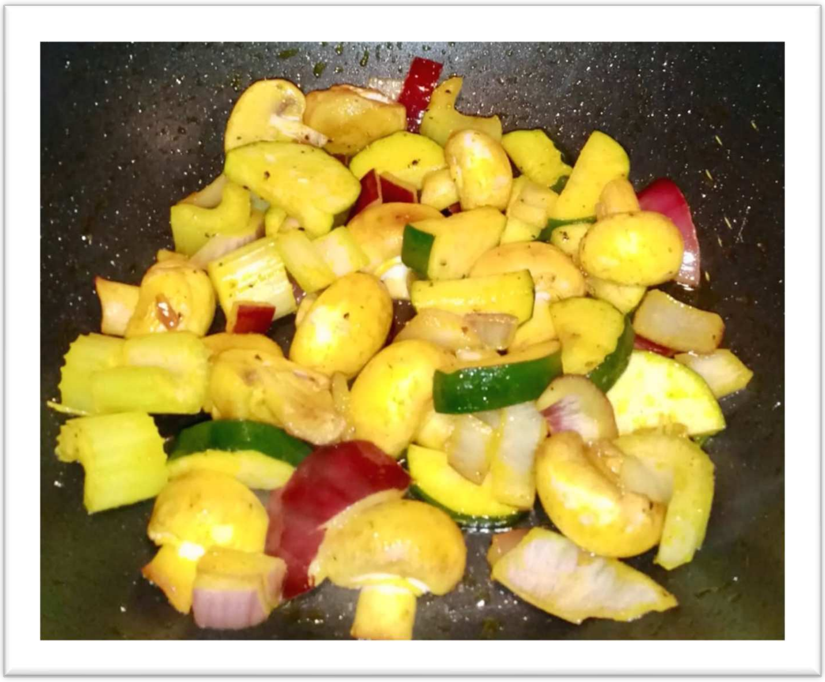

*Figure 2 - Eating colour.*

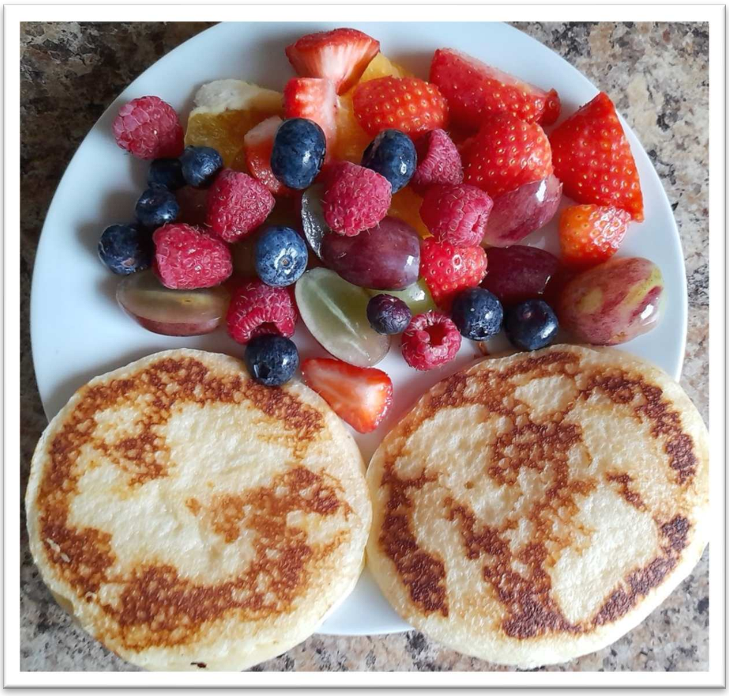

*Figure 3 - What a way to start the day: Homemade pancakes with fresh fruit, beautiful colour and provides me with protein, vitamins and minerals.*

The meaning of nutrition for Irish cancer survivors: A photo voice study

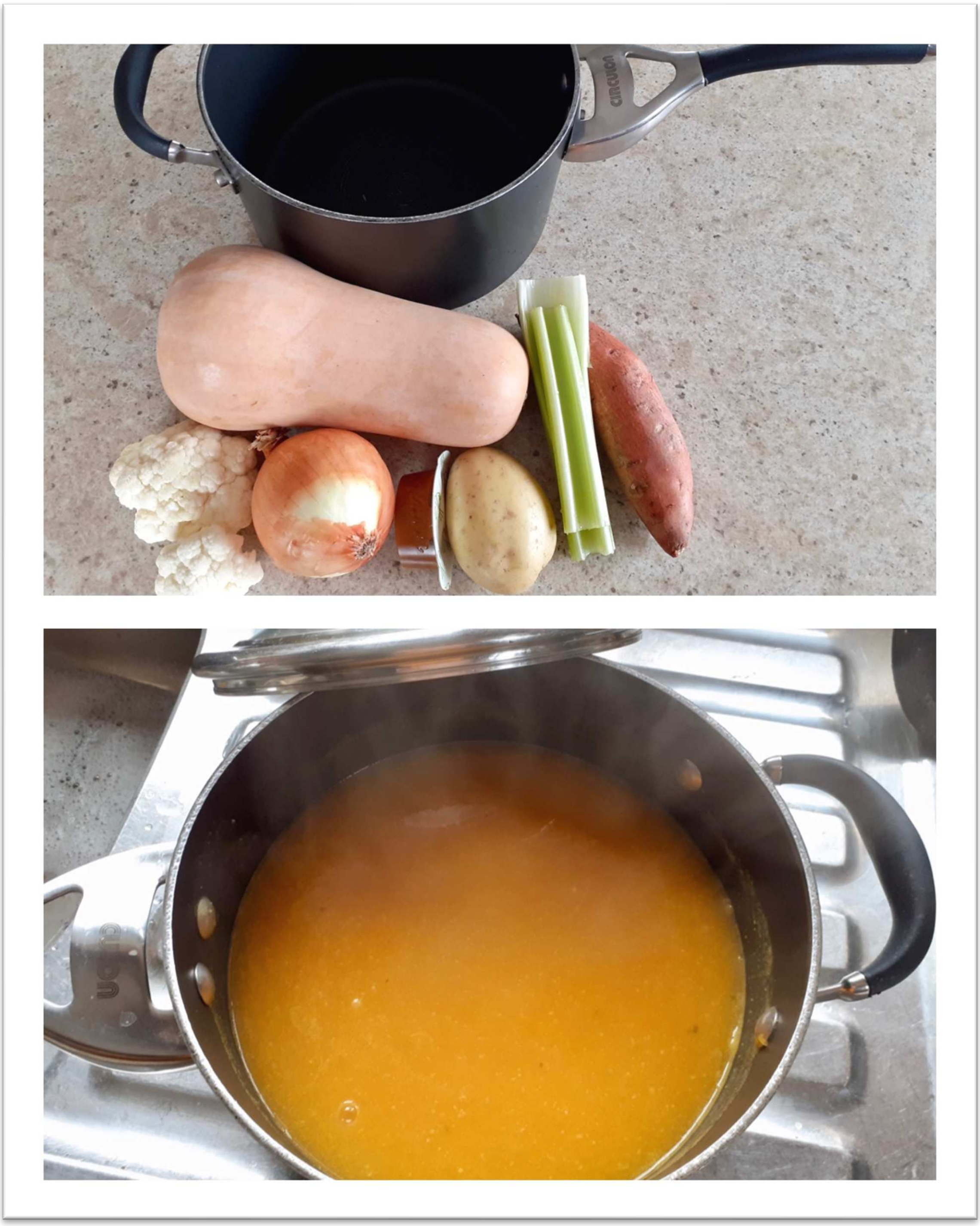

*Figure 4 - Plant a pot When I look at this photo I know my ingredients, no artificial colours or favouring's. Quick, easy.*

The meaning of nutrition for Irish cancer survivors: A photo voice study

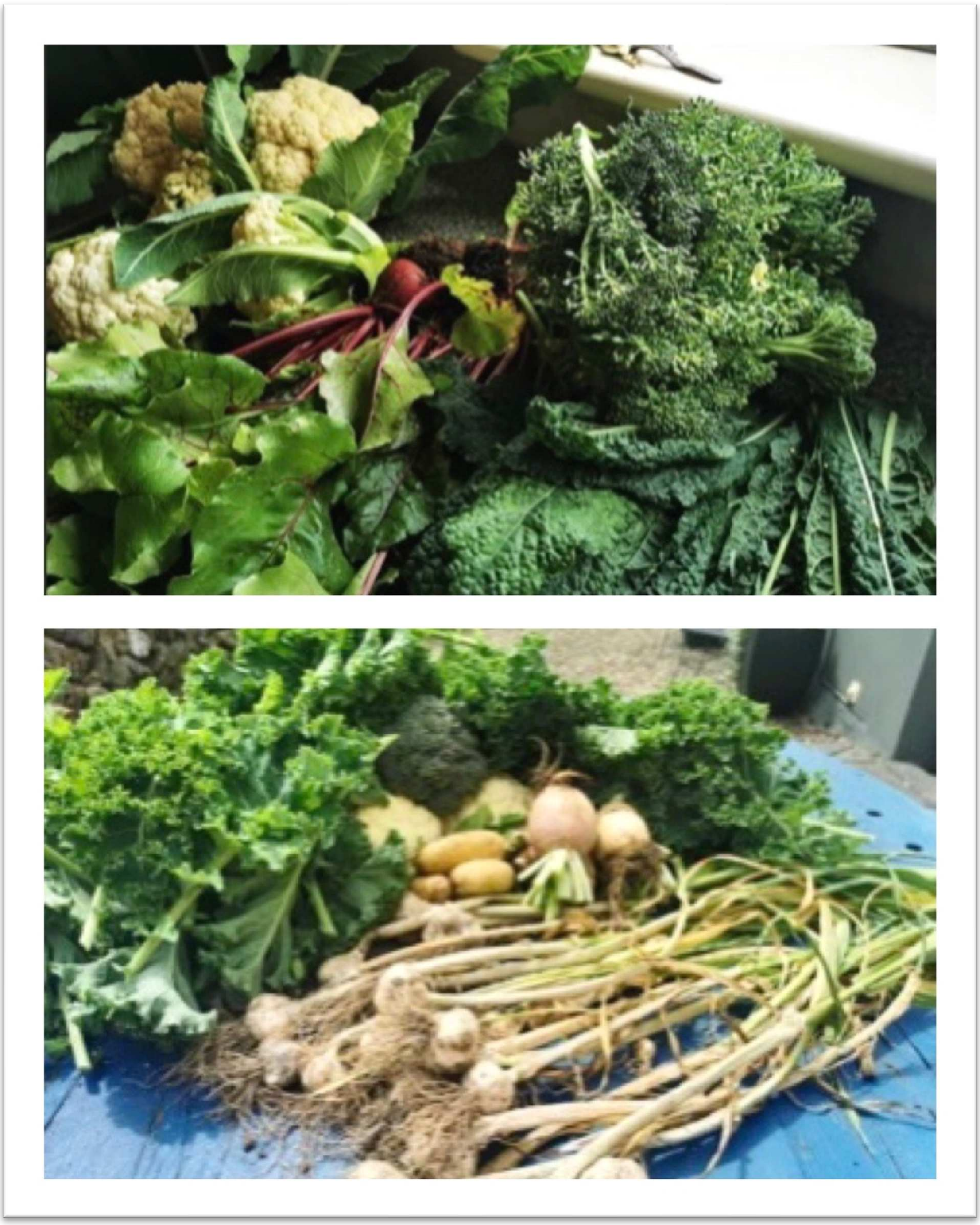

*Figure 5 - Back to basics.*

The meaning of nutrition for Irish cancer survivors: A photo voice study

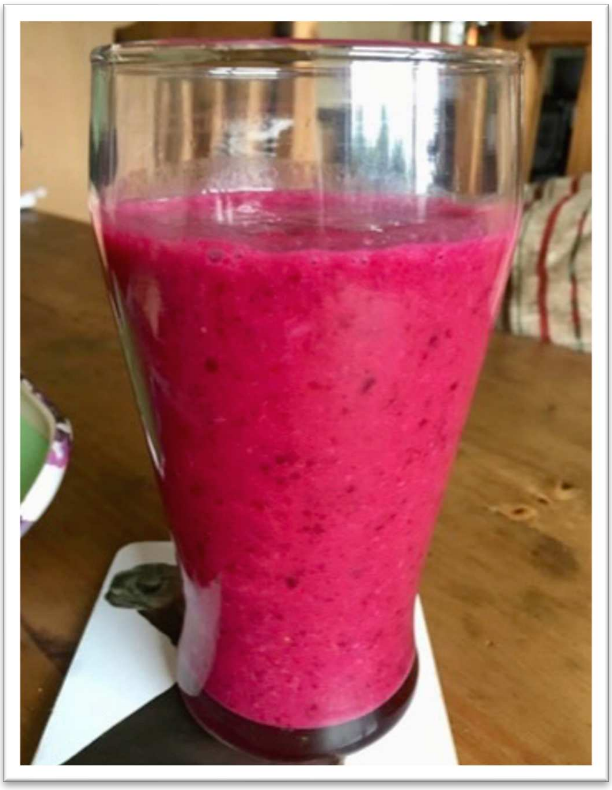

*Figure 6 - All the goodness in a glass.*

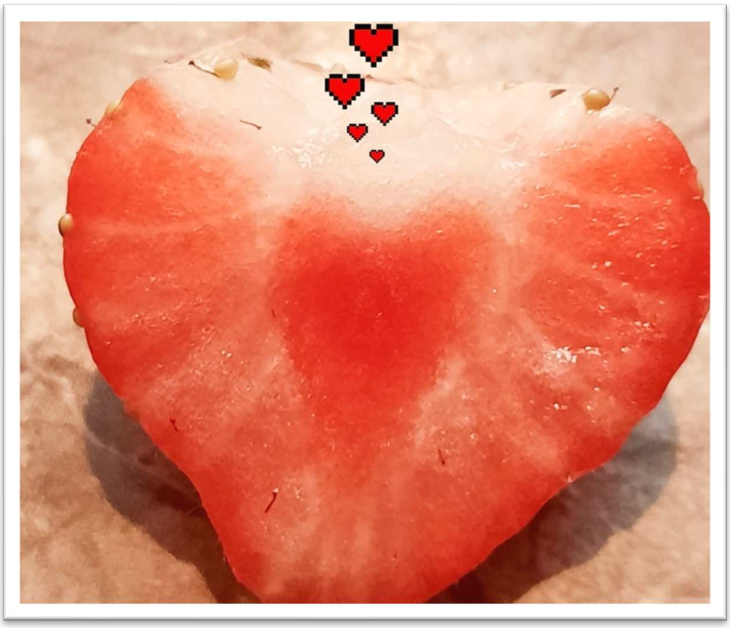

*Figure 7 - The heart of my day.*

The meaning of nutrition for Irish cancer survivors: A photo voice study

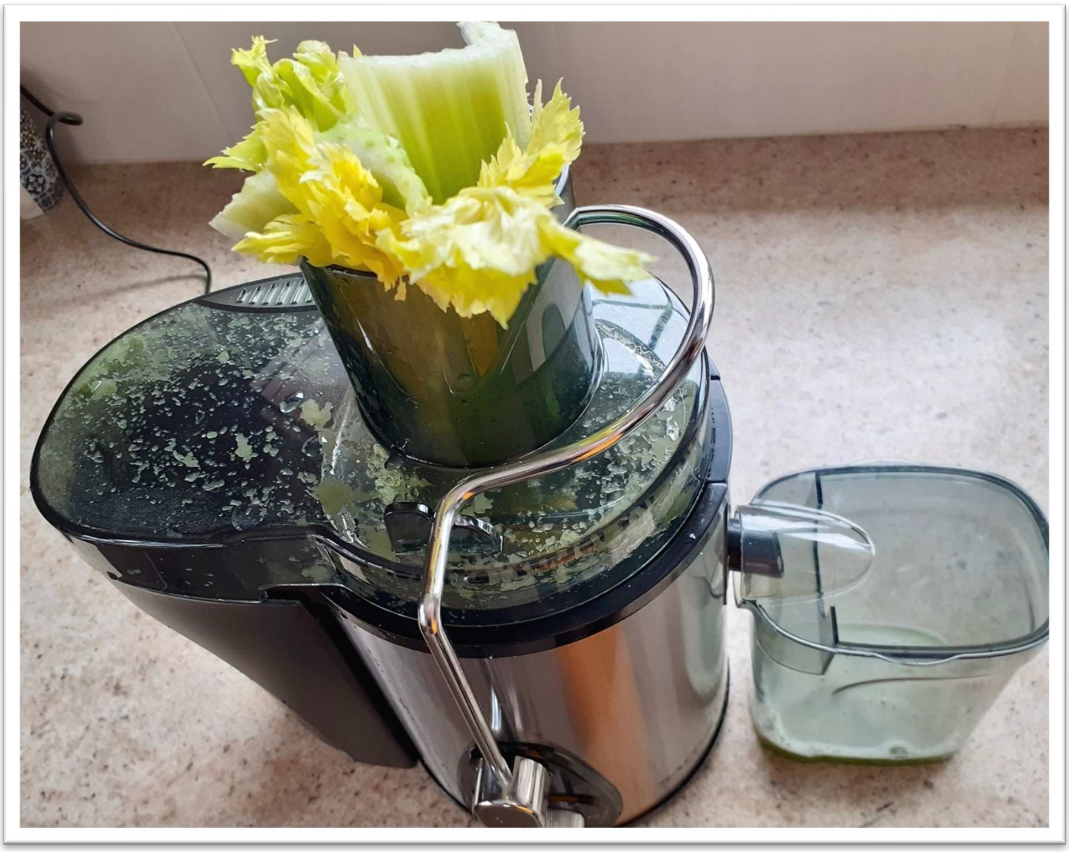

*Figure 8 - Morning Ritual Post treatment each morning I take my celery juice to start the day.*

The meaning of nutrition for Irish cancer survivors: A photo voice study

## Theme 2: Building Blocks. Be Informed.

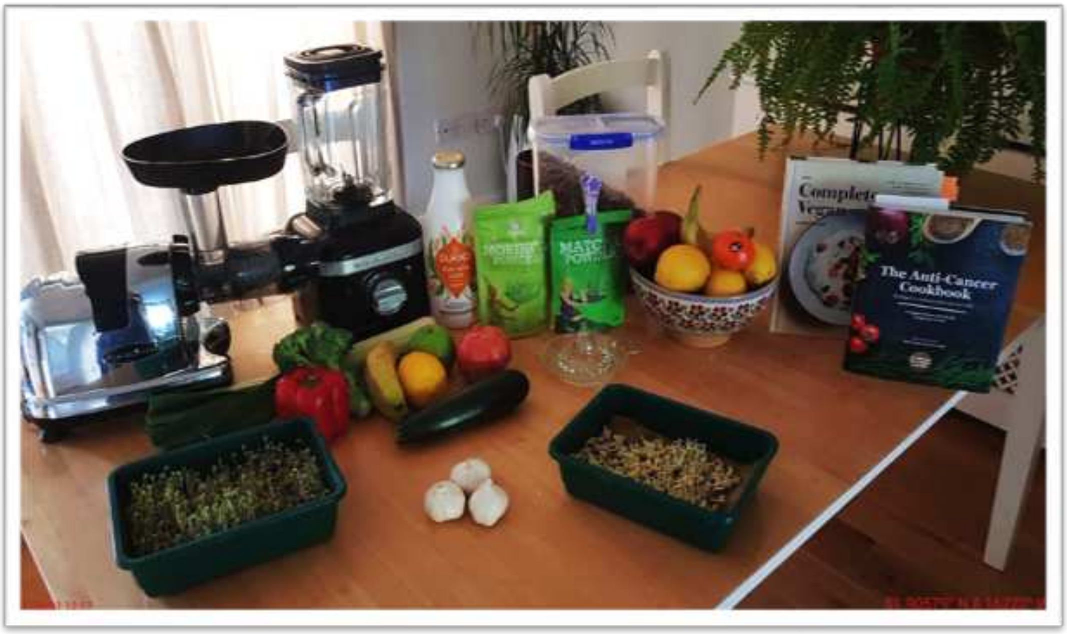


*Figure 9 - New Diet & Nutrition*

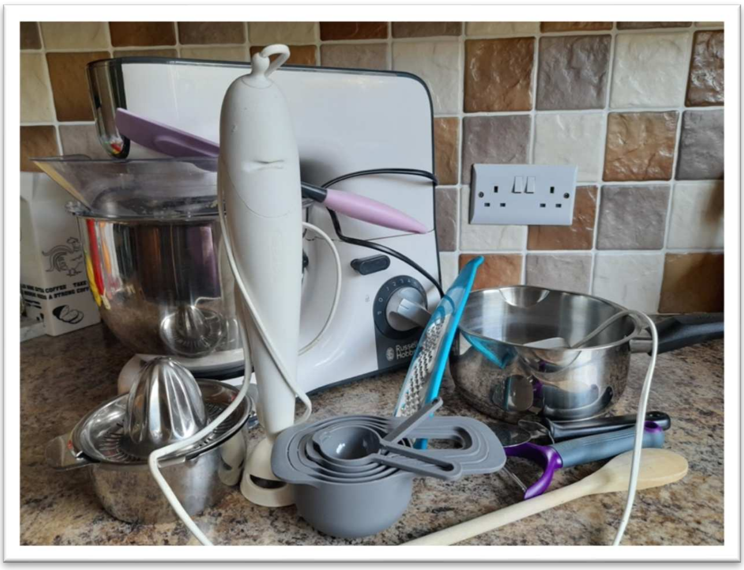

*Figure 10 - Good tools. Good foods.*

The meaning of nutrition for Irish cancer survivors: A photo voice study

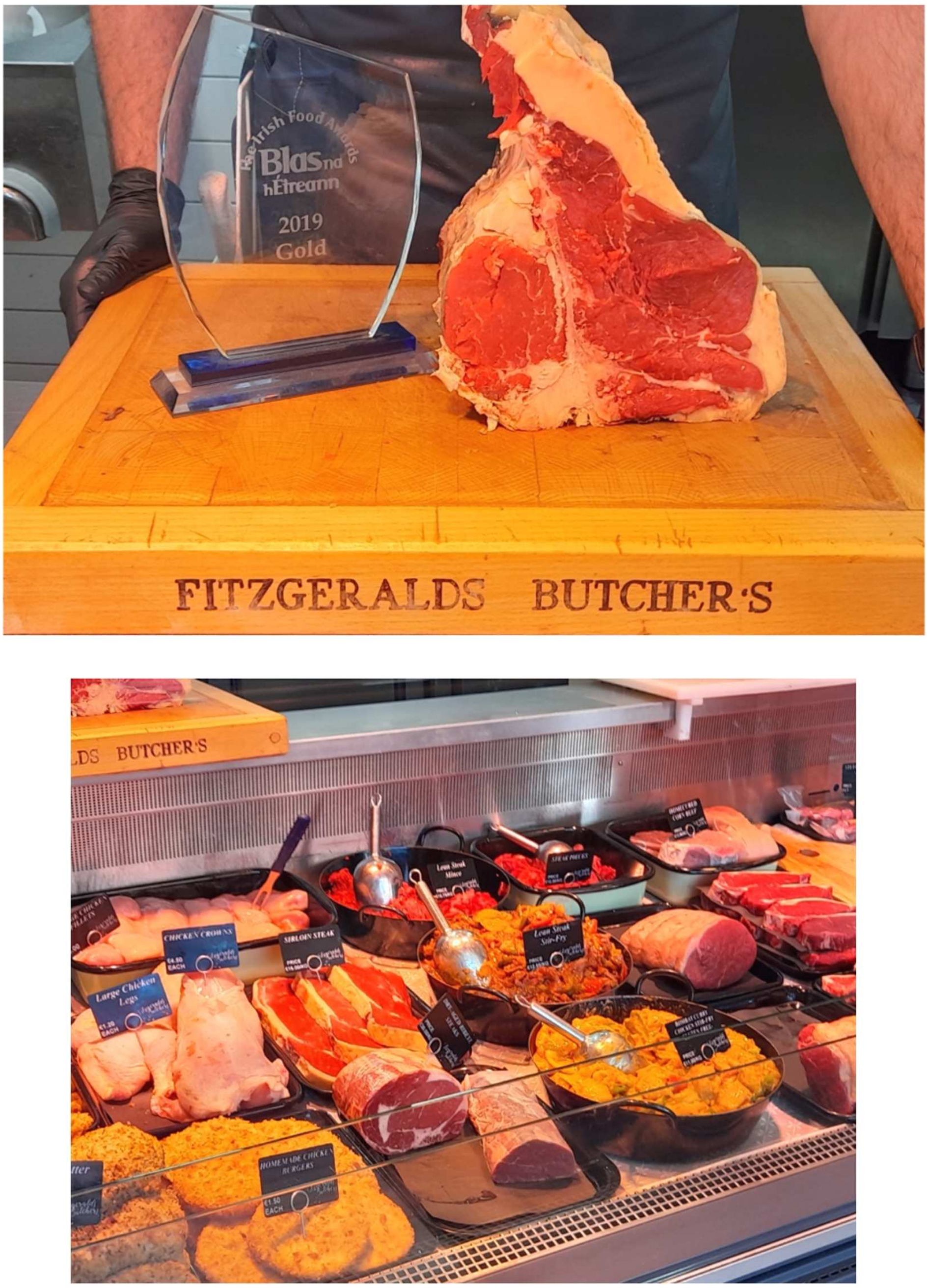


*Figure 11 - Locally sourced: Support local business. It not only reduces my carbon footprint but builds a trustworthy relationship with my local butcher, who can advise on how to store, prepare and cook meat.*

The meaning of nutrition for Irish cancer survivors: A photo voice study

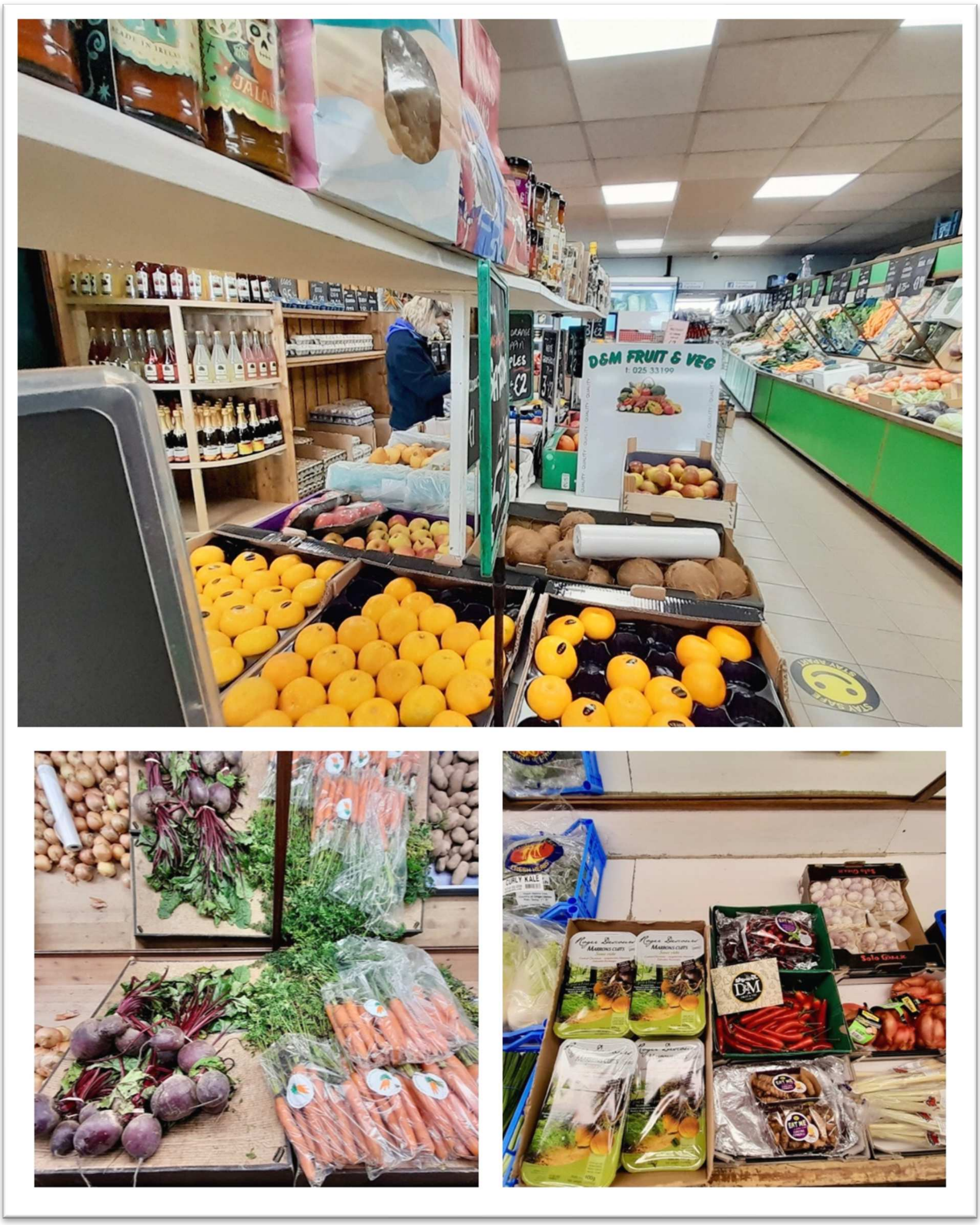


*Figure 12 - My little oasis: I love going into Josephine because her selection of fruit and vegetables is always so fresh and full of colour. I can ask Josephine the best way of incorporating seasonal fruit and vegetables into my diet.*

The meaning of nutrition for Irish cancer survivors: A photo voice study

## Theme 3: Be kind to yourself

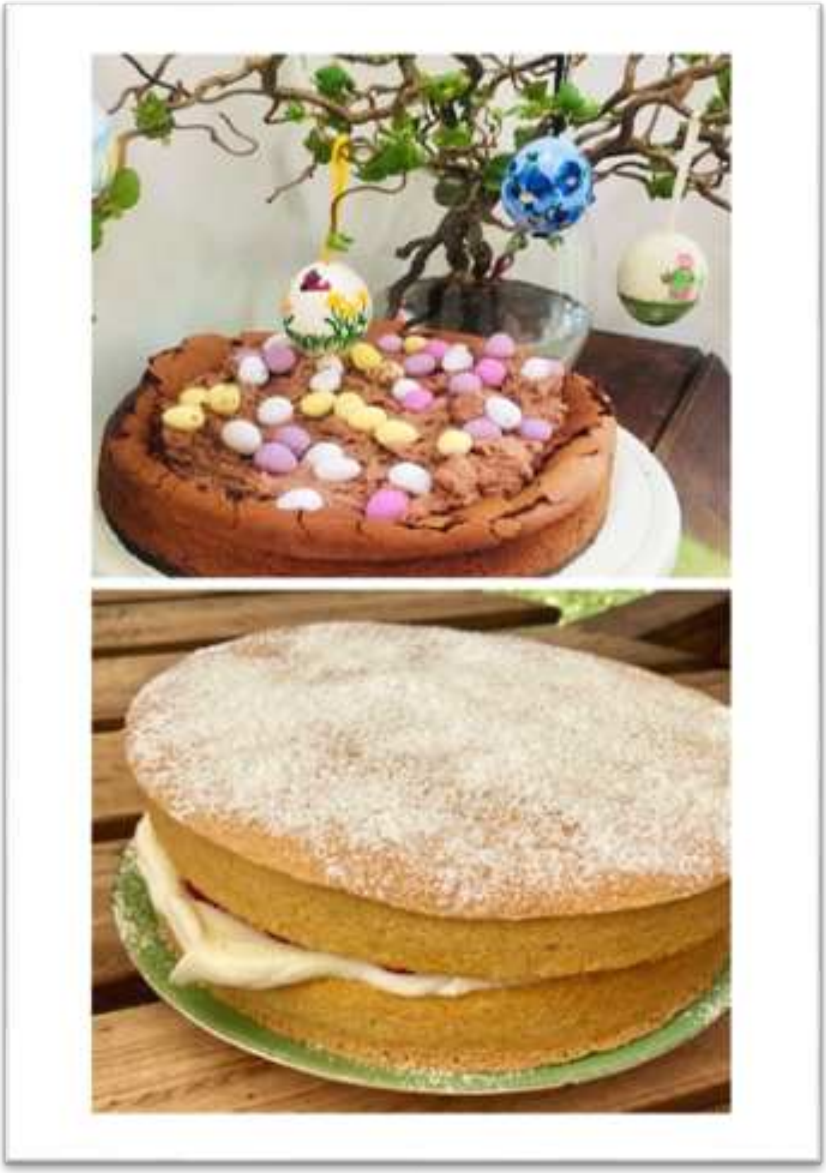

*Figure 13 - We celebrate every milestone and festival with cake!*

The meaning of nutrition for Irish cancer survivors: A photo voice study

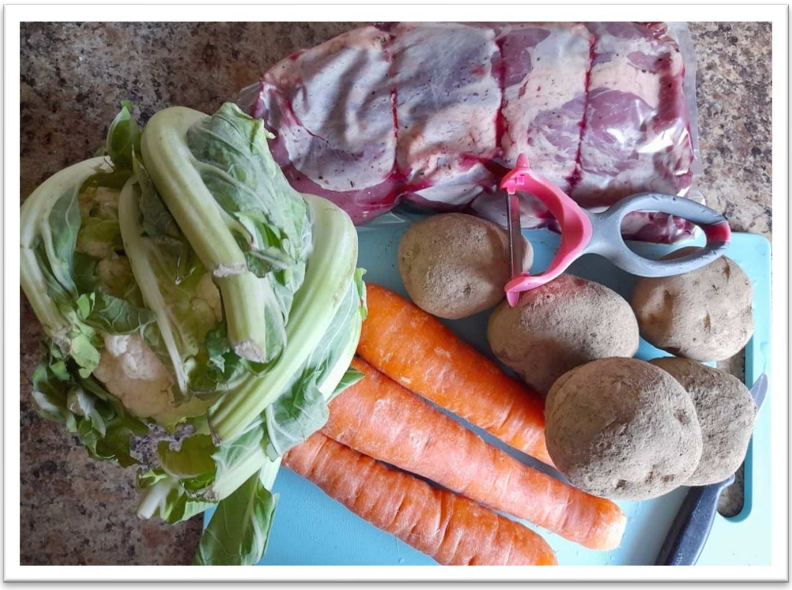

*Figure 14 - Family: A dinner for family get together a good old fashioned roast.*

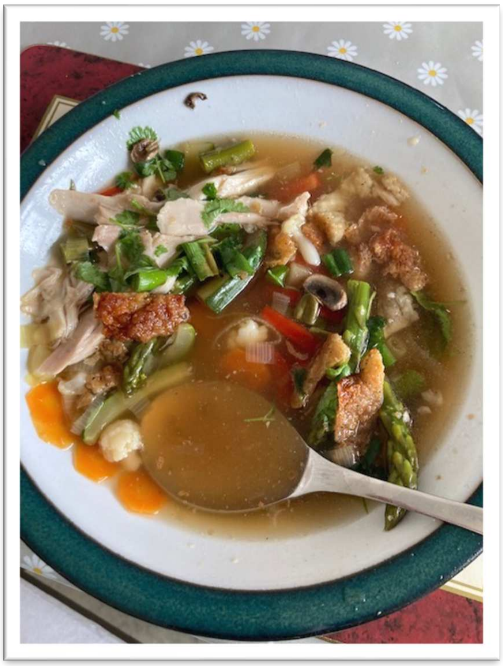

*Figure 15 - This was the last meal I cooked my mother before she died. She came for lunch during her treatment and was struggling again with eating. But she loved this soup and loved how pretty it looked.*

The meaning of nutrition for Irish cancer survivors: A photo voice study

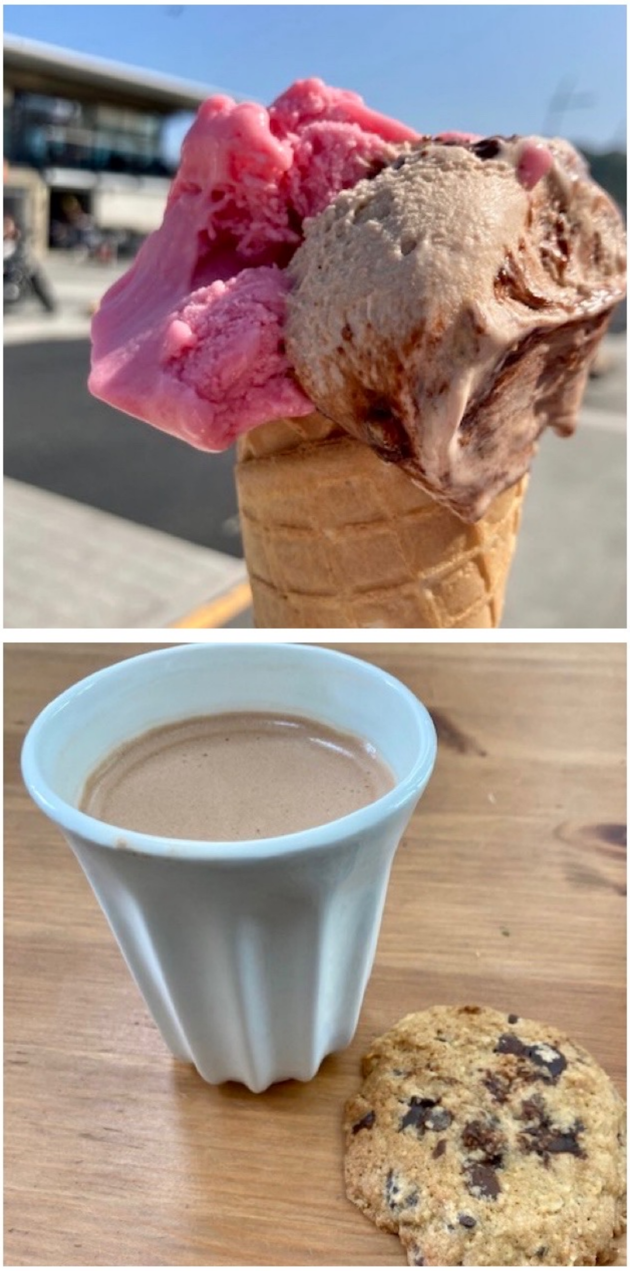

*Figure 16 - Time out.*

The meaning of nutrition for Irish cancer survivors: A photo voice study

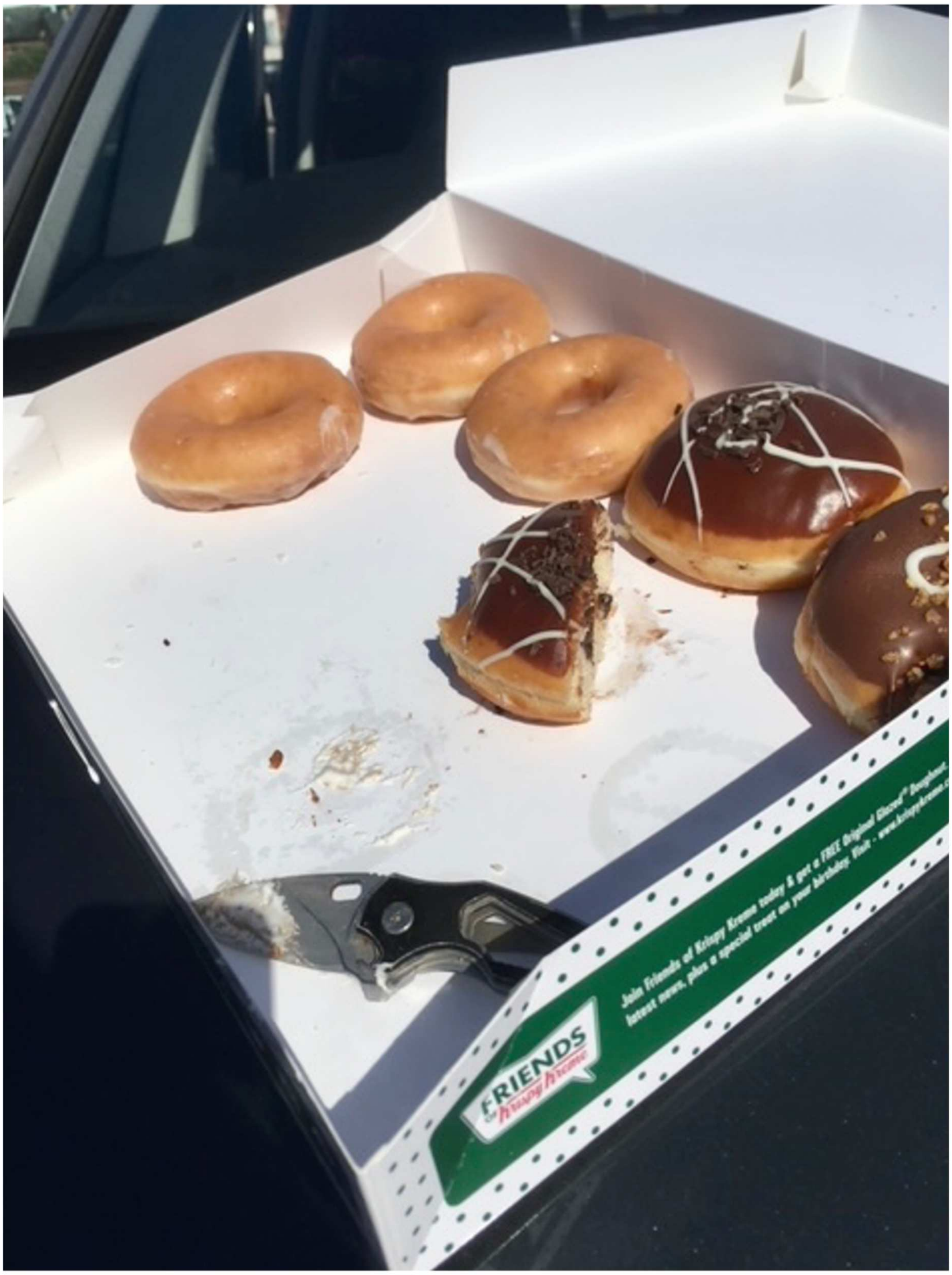


*Figure 17 - Post celebration after my last radiotherapy - only had to drive across England to a Krispy Kreme!*

The meaning of nutrition for Irish cancer survivors: A photo voice study

## Theme 4: Post-Treatment Healing Changes

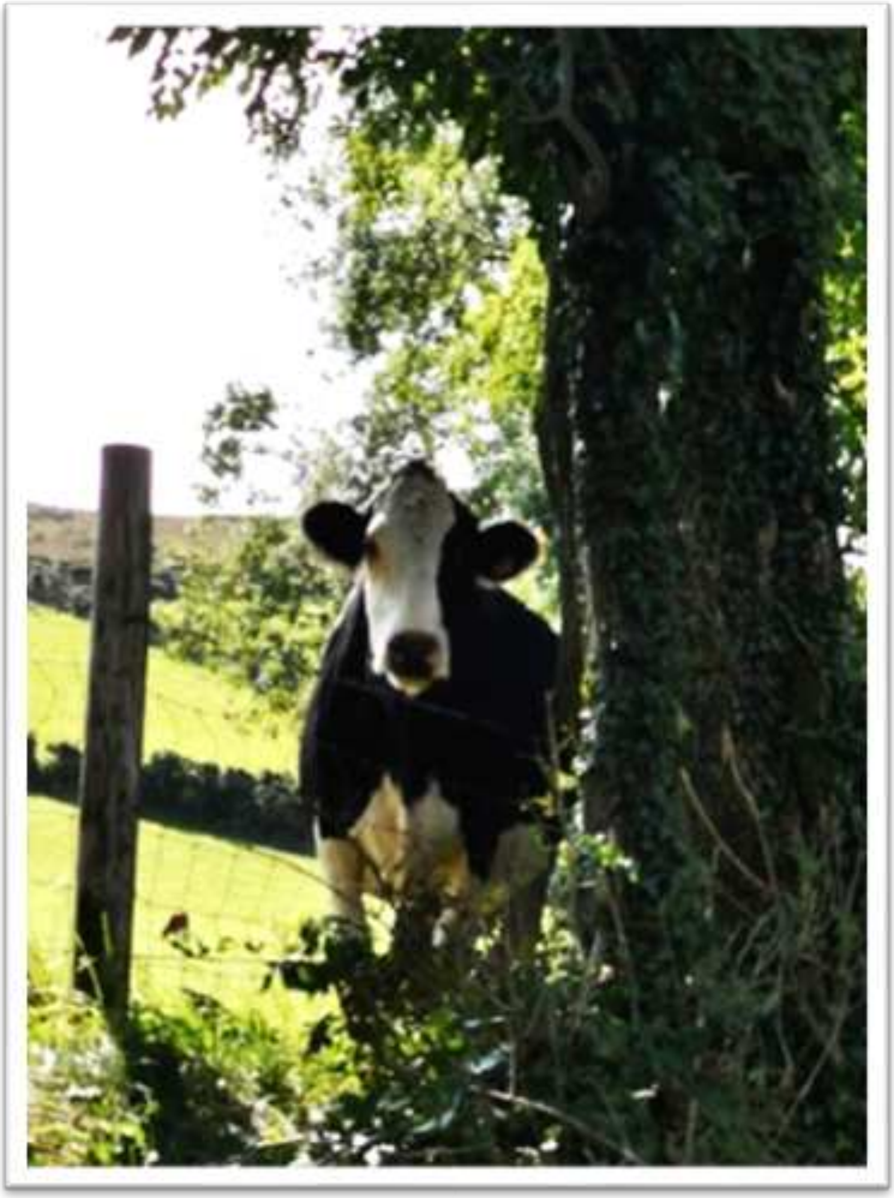

*Figure 18 - This is the only dairy I see.*

The meaning of nutrition for Irish cancer survivors: A photo voice study

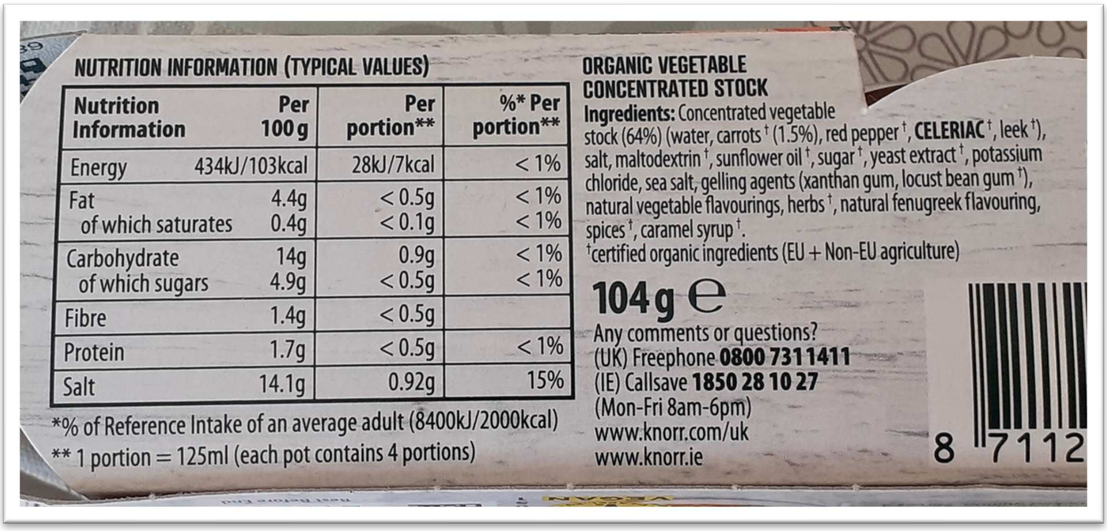


*Figure 19 - If you can't read it don't eat it.*

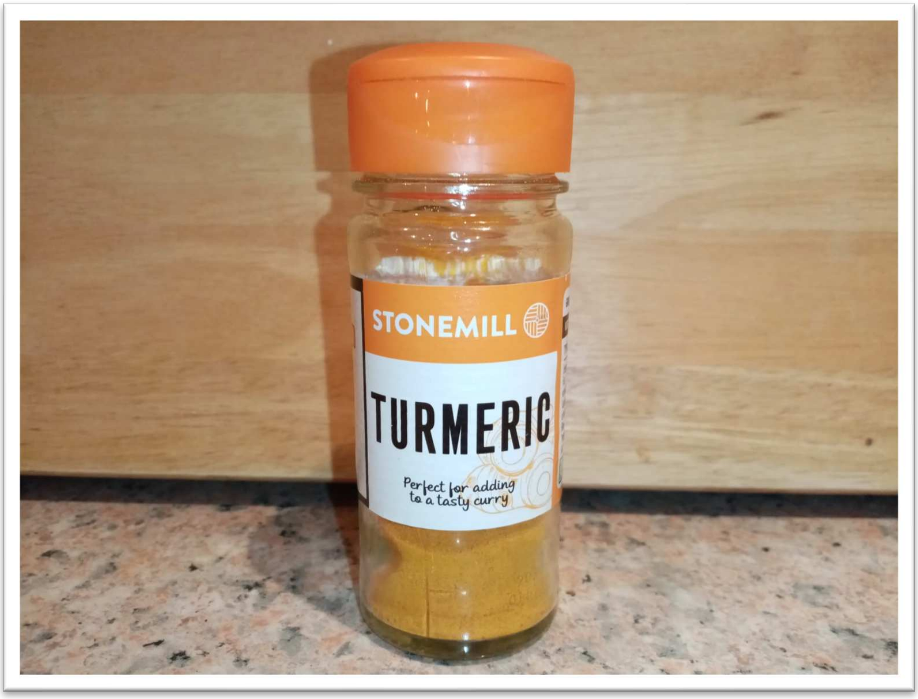


*Figure 20 – Anti-inflammatory*

The meaning of nutrition for Irish cancer survivors: A photo voice study

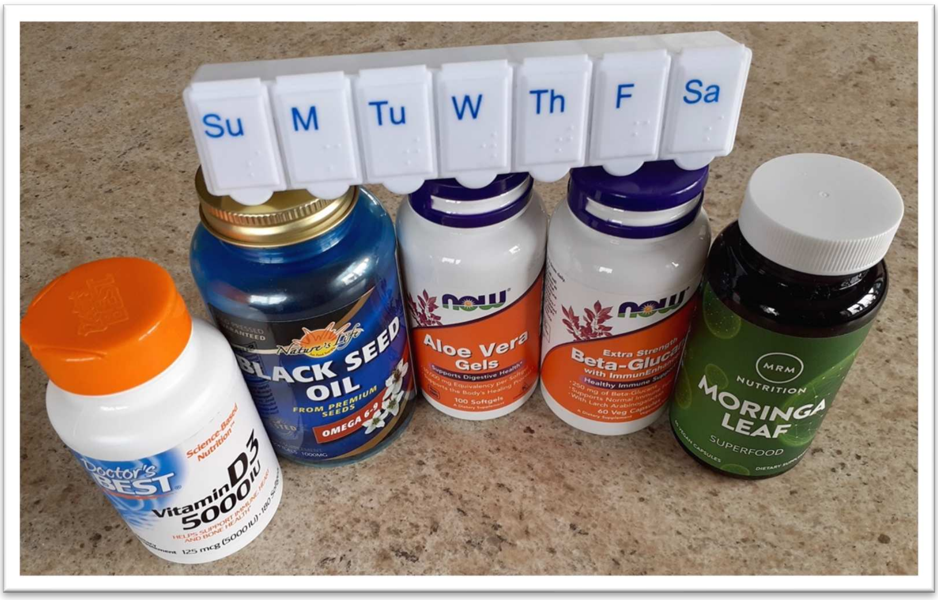


*Figure 21 - Post treatment routine.*

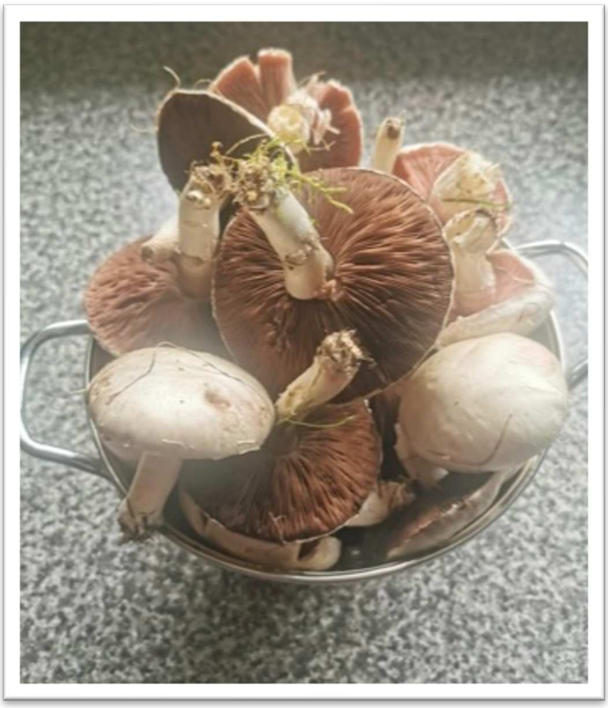

*Figure 22 - Anti-inflammatory properties*

The meaning of nutrition for Irish cancer survivors: A photo voice study

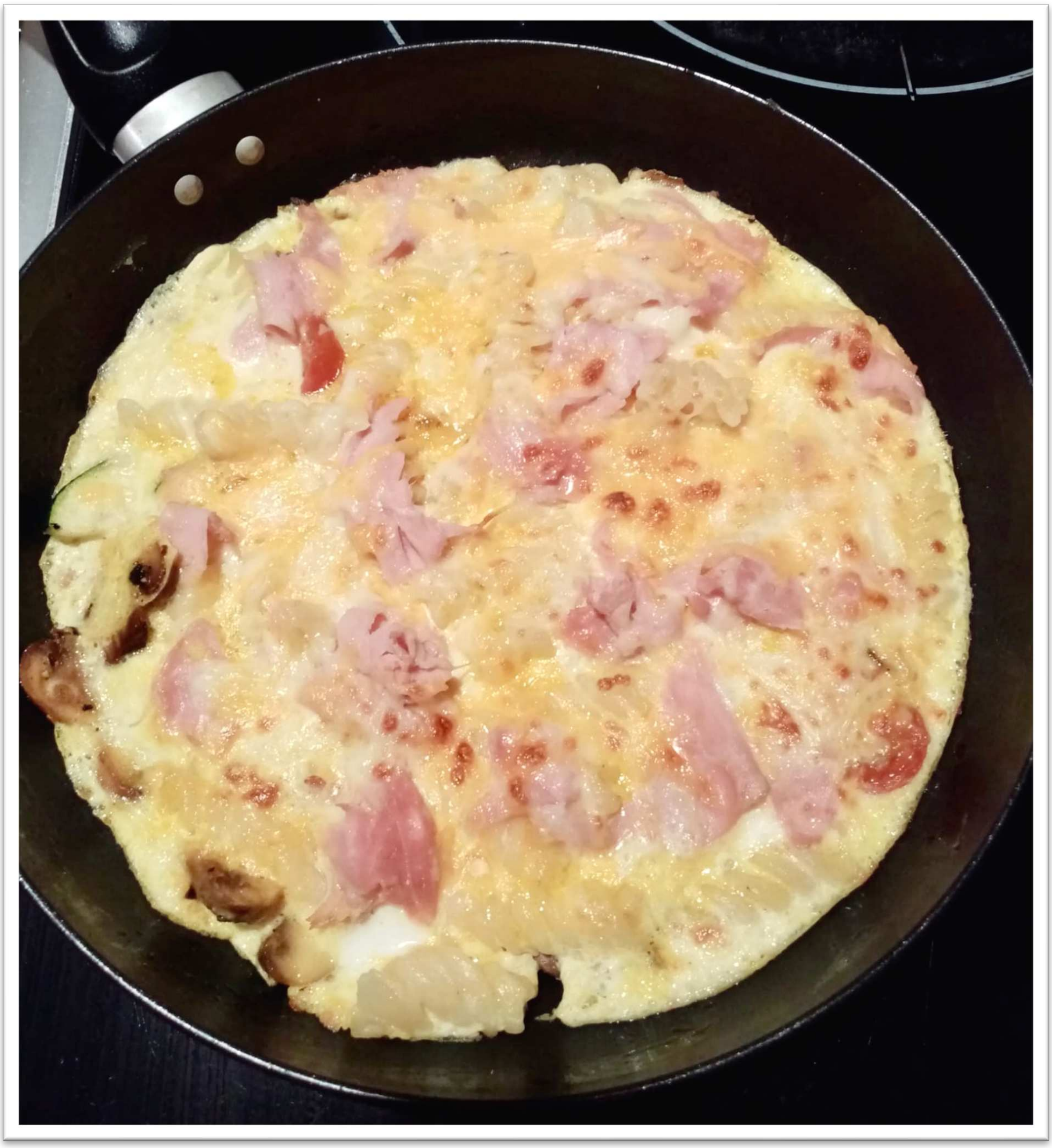

*Figure 23 - Easy prep, minimum time, tasty, nourishing and gentle on poor tummy.*

The meaning of nutrition for Irish cancer survivors: A photo voice study

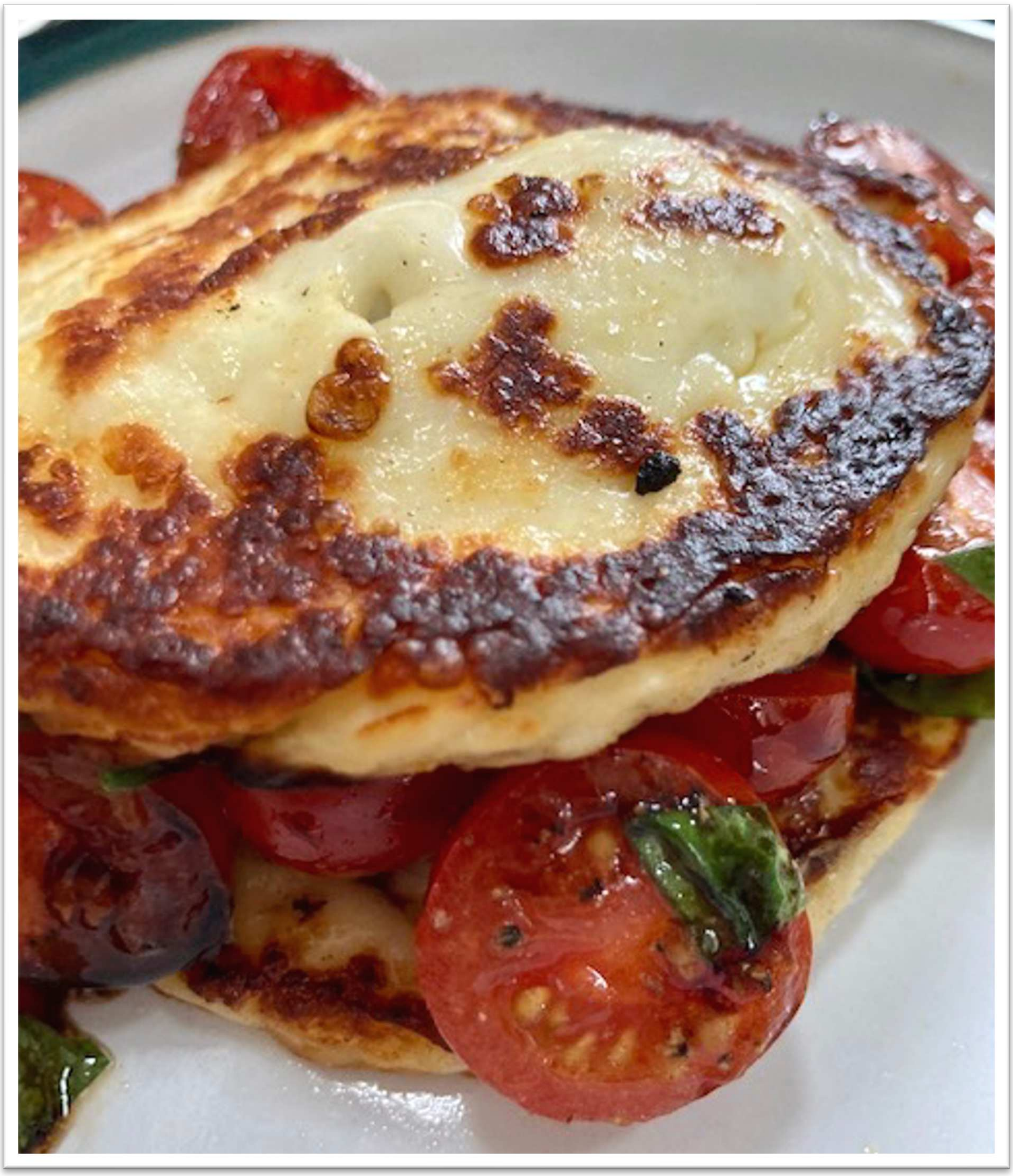

*Figure 24 - The time my photo went viral on Twitter No chilli but fresh homegrown tomatoes - my take on @nigella_lawson halloumi & chilli*

The meaning of nutrition for Irish cancer survivors: A photo voice study

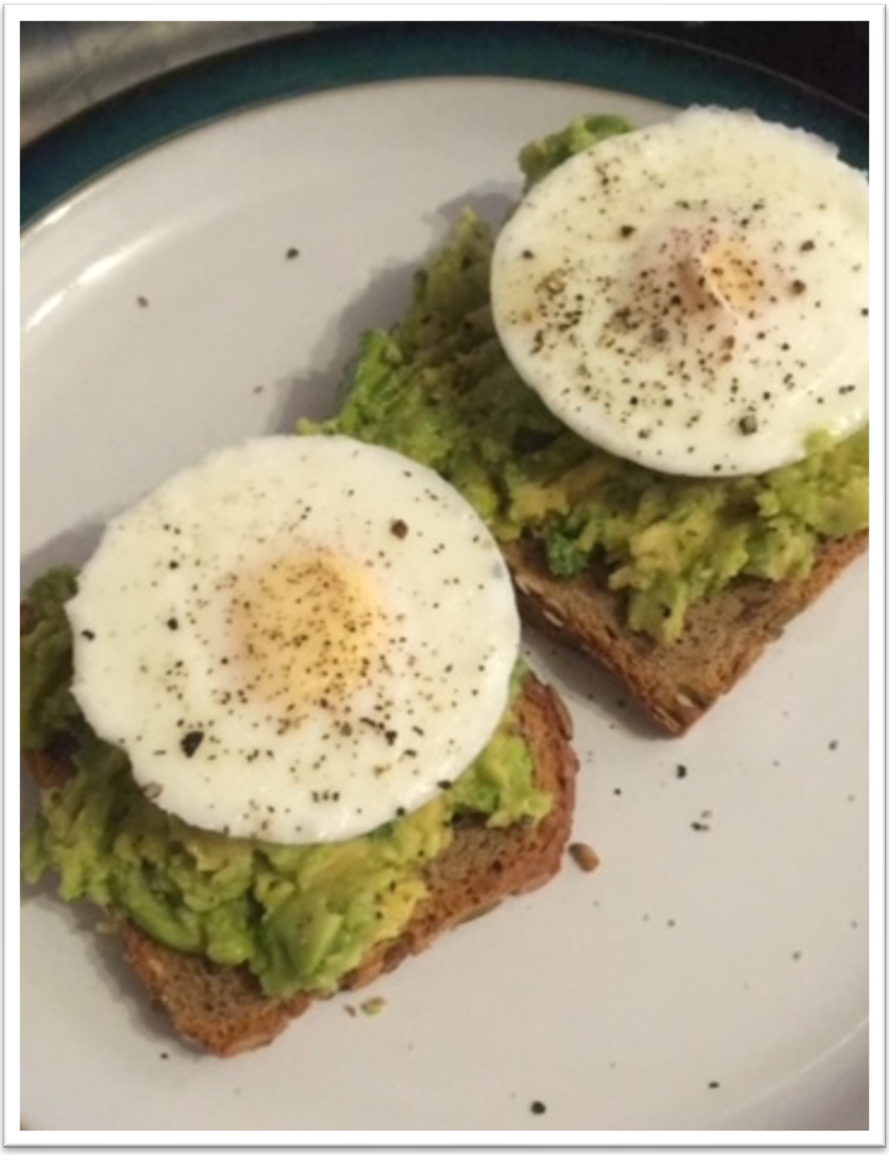

*Figure 25 - Boobies!!!*

The meaning of nutrition for Irish cancer survivors: A photo voice study

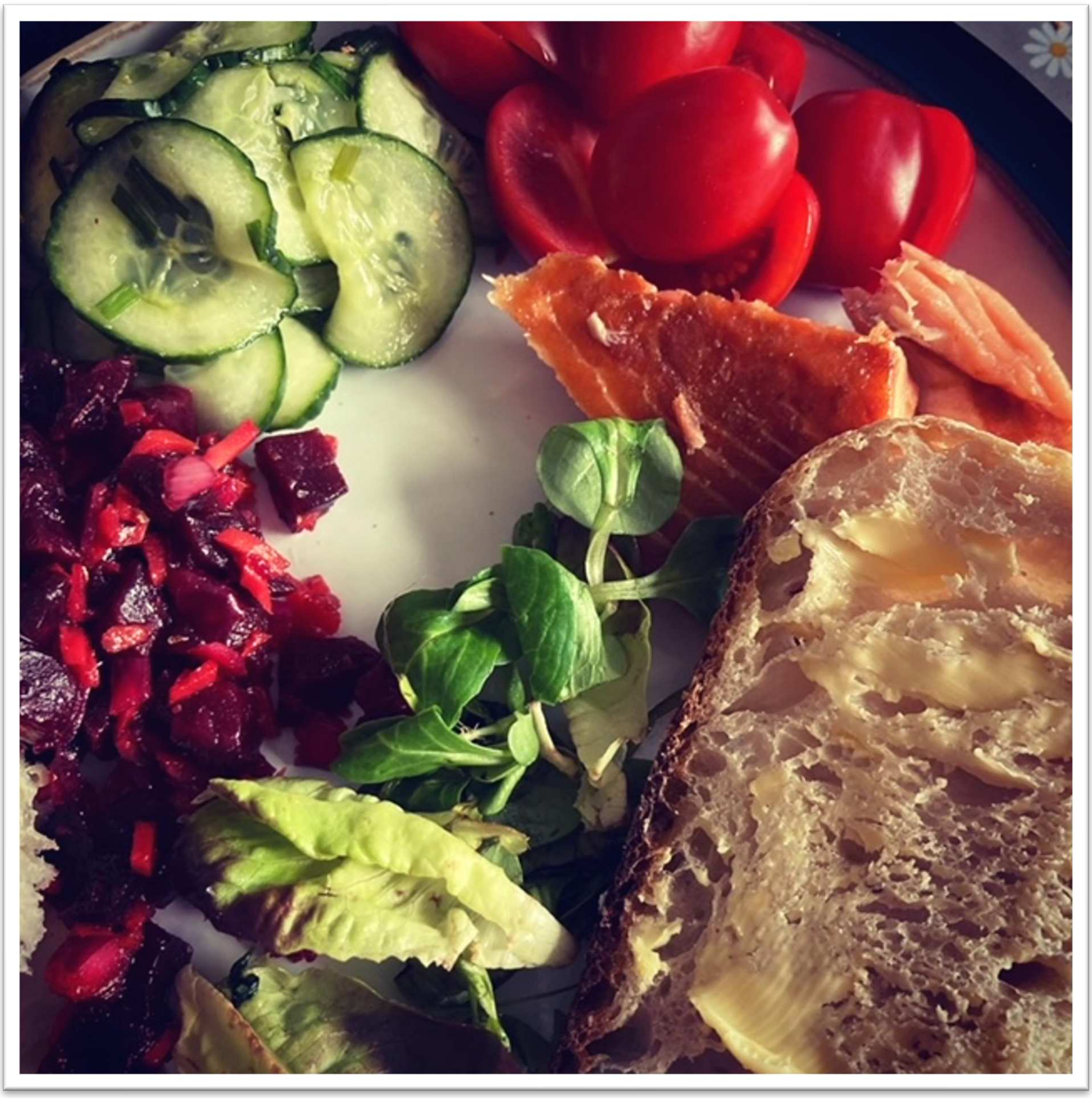

*Figure 26 - I love my food, my biggest fear before chemo was losing my sense of taste. I never took food for granted but when faced with losing my sense of taste, I realised how important food was to me. My memories are linked to food! I love family recipes, recreating meals out. I have a huge library of cookery books, I mainly follow chefs and food writers on Instagram and Twitter. I love to keep a record of my food and love to celebrate my food.*

The meaning of nutrition for Irish cancer survivors: A photo voice study

## Theme 5: Chemo Rituals

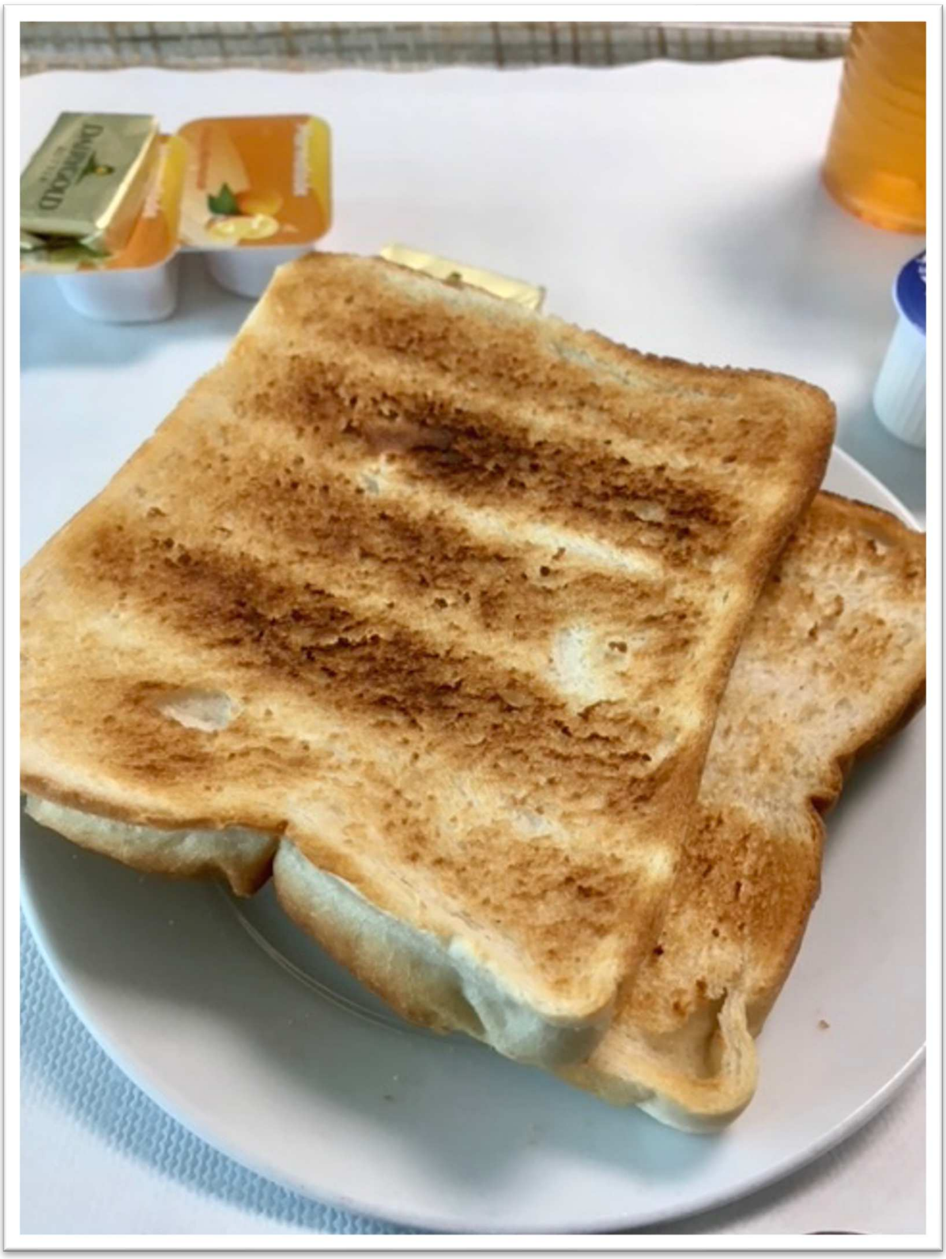


*Figure 27 - Chemo Breakfast.*

The meaning of nutrition for Irish cancer survivors: A photo voice study

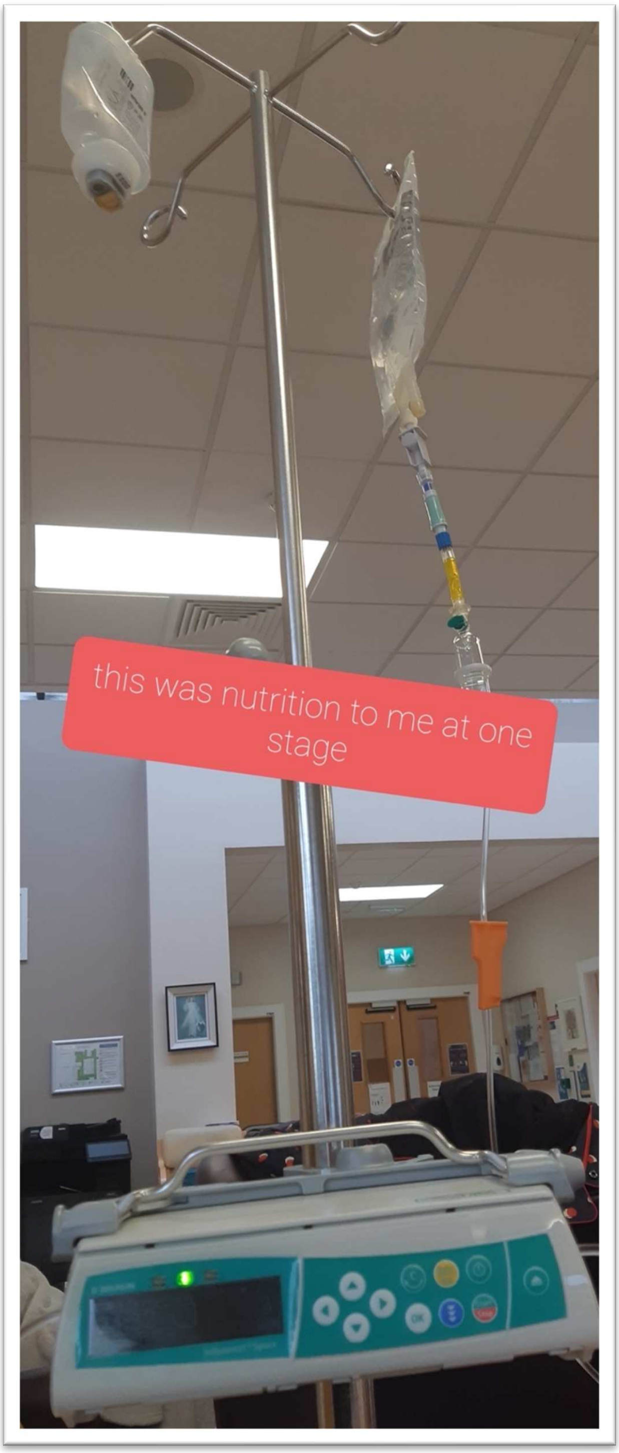


*Figure 28 - This was nutrition for me at one stage.*

The meaning of nutrition for Irish cancer survivors: A photo voice study

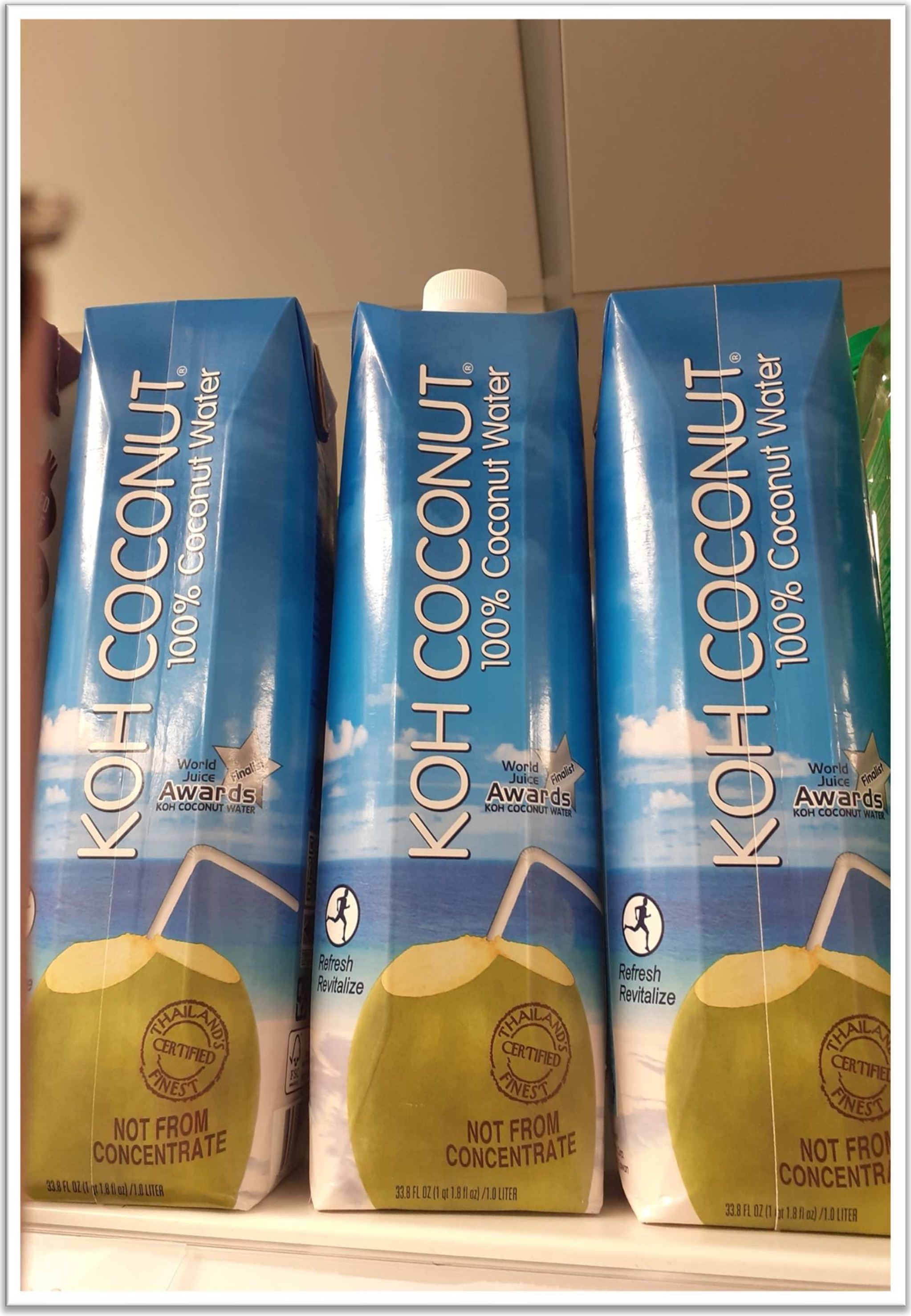


*Figure 29 - The Coconut Water .. this represented for me that my bloods were always right before my Chemo, there is a lot of nutrition in it and throughout chemo I drank many litres.*

The meaning of nutrition for Irish cancer survivors: A photo voice study

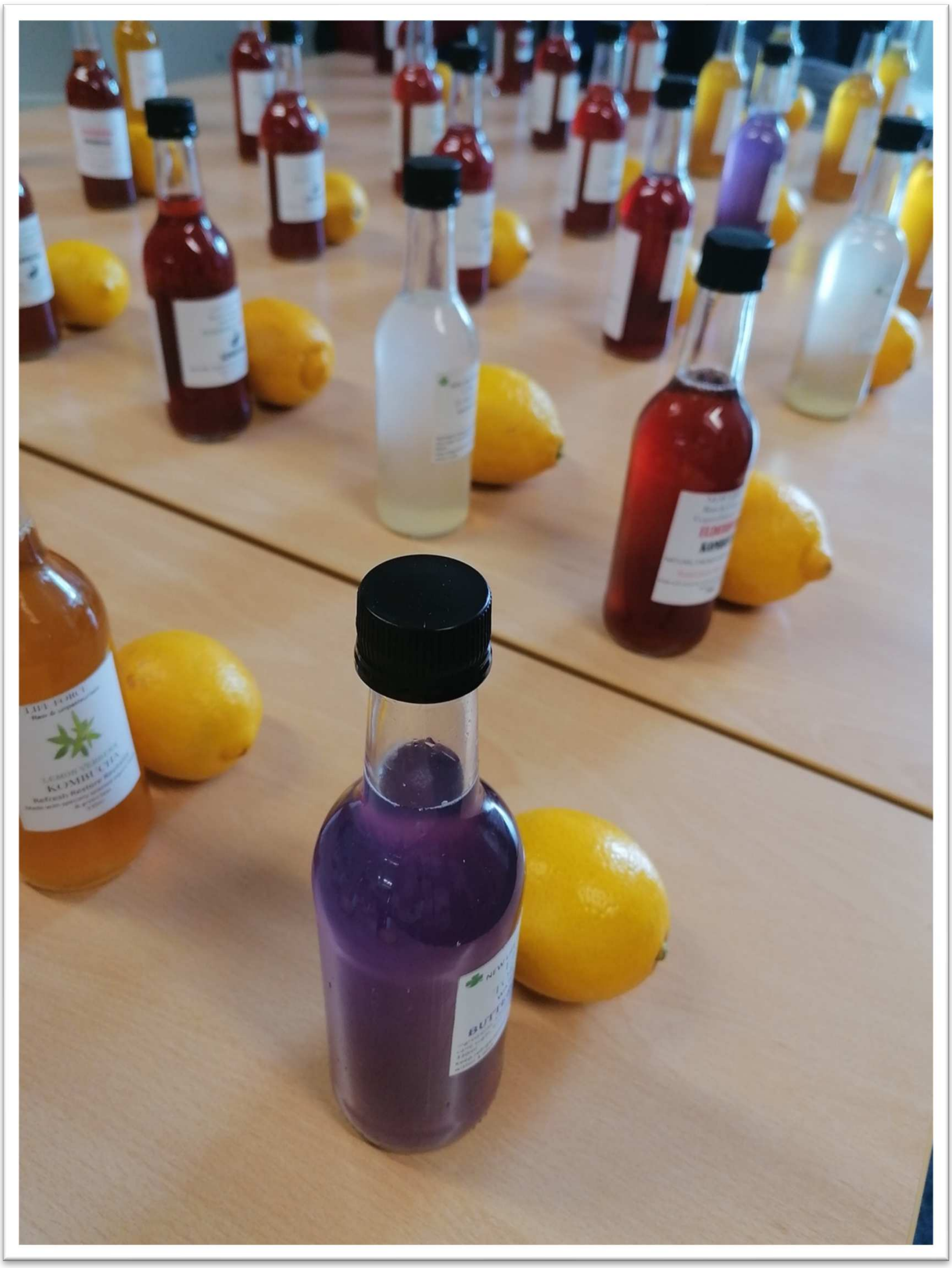

*Figure 30 - Buttery peas - Our cancer support groups response to the pandemic. Water Kefir made from Buttery peas, organic honey and lemon with sourdough bread and soup. This was packed weekly and sent to our 100 plus members. Made from organic ingredients from our allotment. When I took this picture, I imagined the transformation of a caterpillar to a butterfly, and the healing qualities of an army of these bottles!*

The meaning of nutrition for Irish cancer survivors: A photo voice study

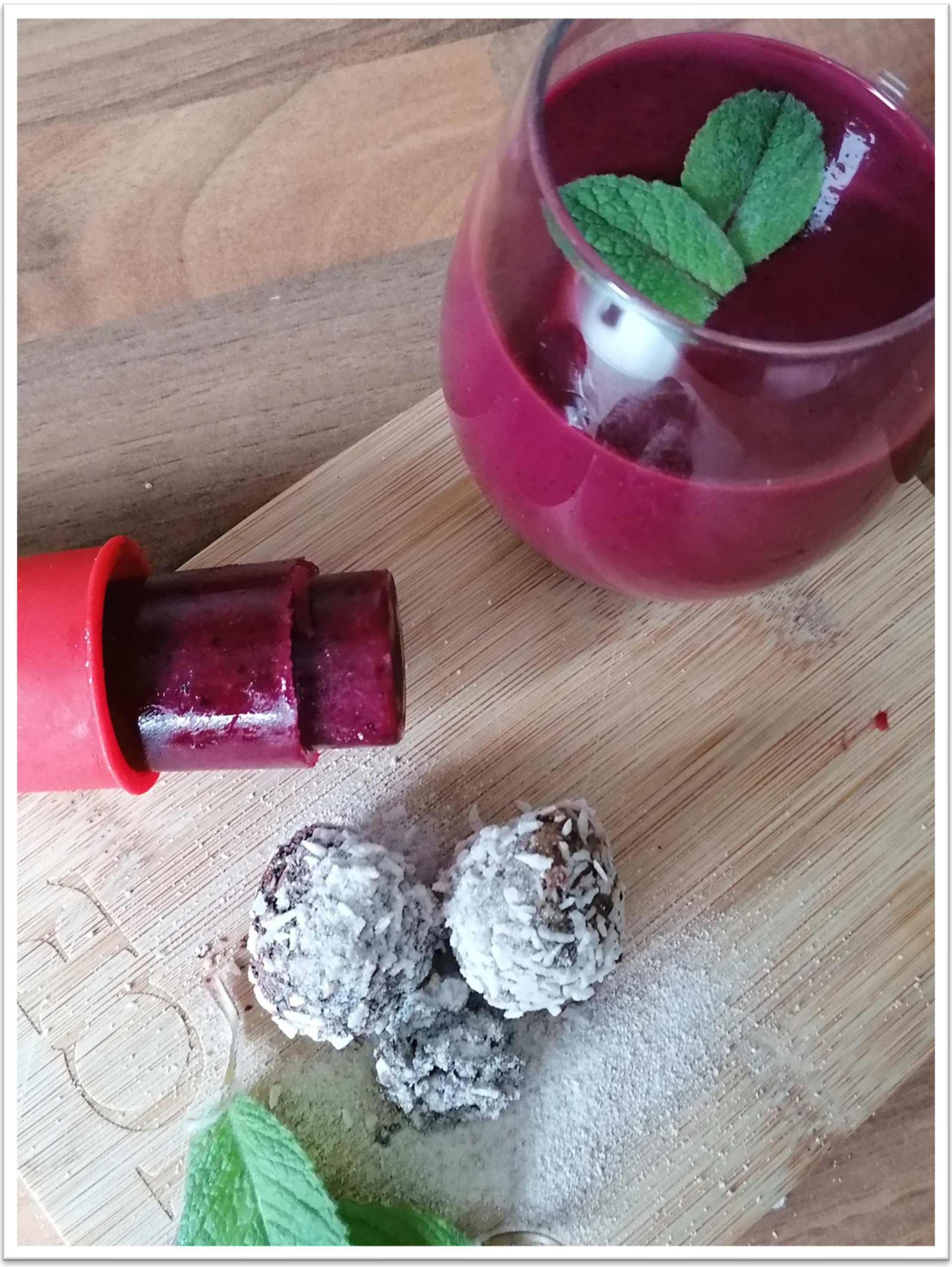

*Figure 31 - Beetroot juice and Protein Balls - the foods no one ever recommended on a cancer diet!*

The meaning of nutrition for Irish cancer survivors: A photo voice study

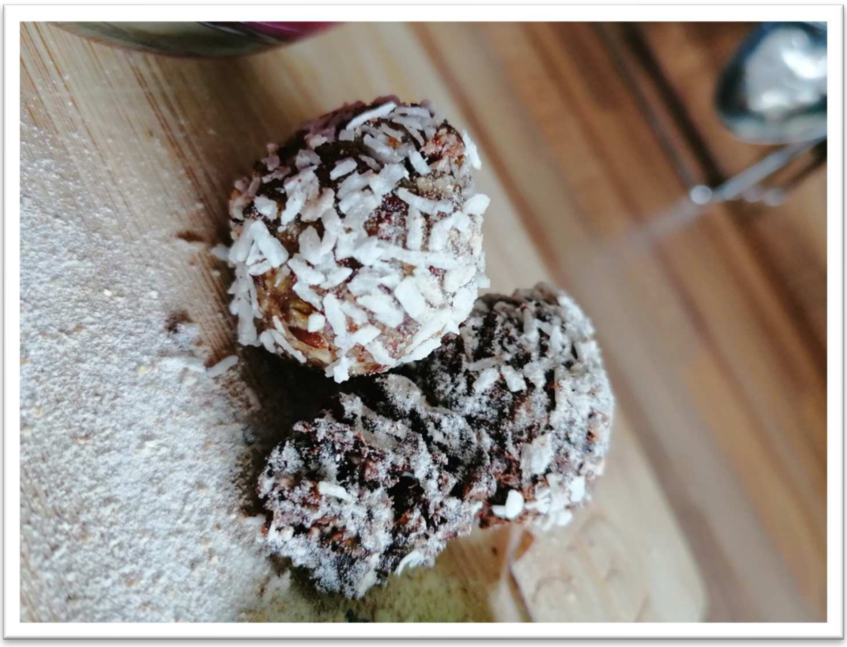

*Figure 32 - Protein Power – hand- made organic cocoa nibs and coconut energy balls. I lived on these for energy throughout treatment and they always looked so appetising. Looking at them reminds me of time spent in the kitchen, and the drive I had to eat myself well again.*

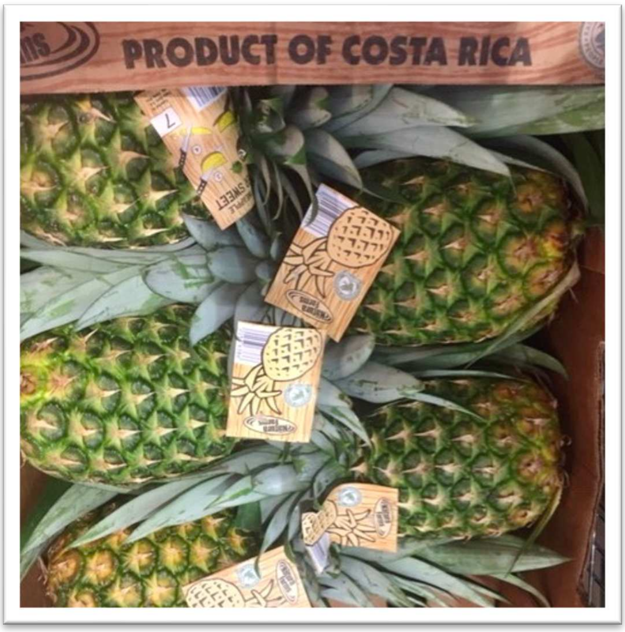


*Figure 33 - Bromelain - aids the healing process.*

The meaning of nutrition for Irish cancer survivors: A photo voice study

Theme 6: Food for the Soul – Healthy Mind. Healthy Body.

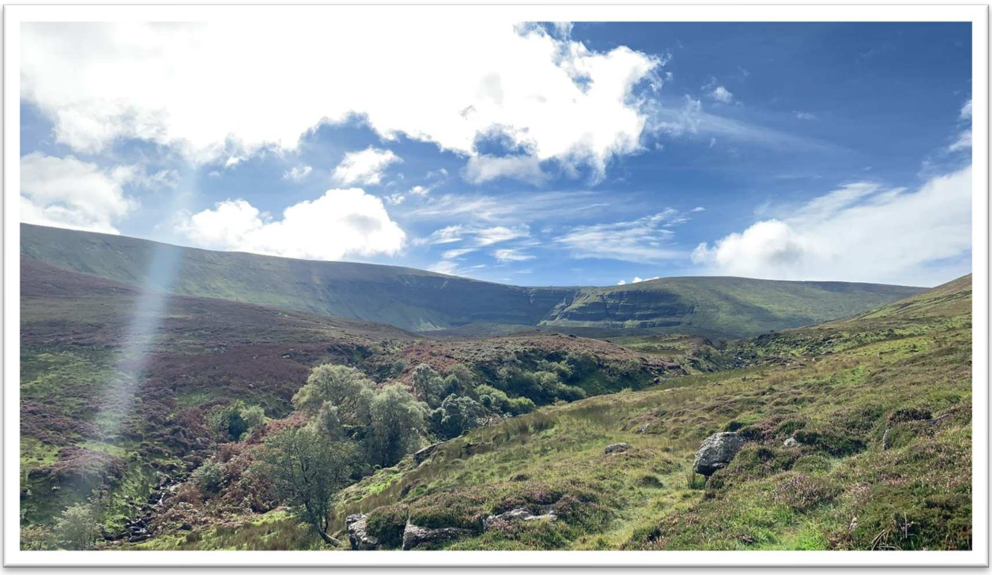

*Figure 34 - Exercise & Environment*

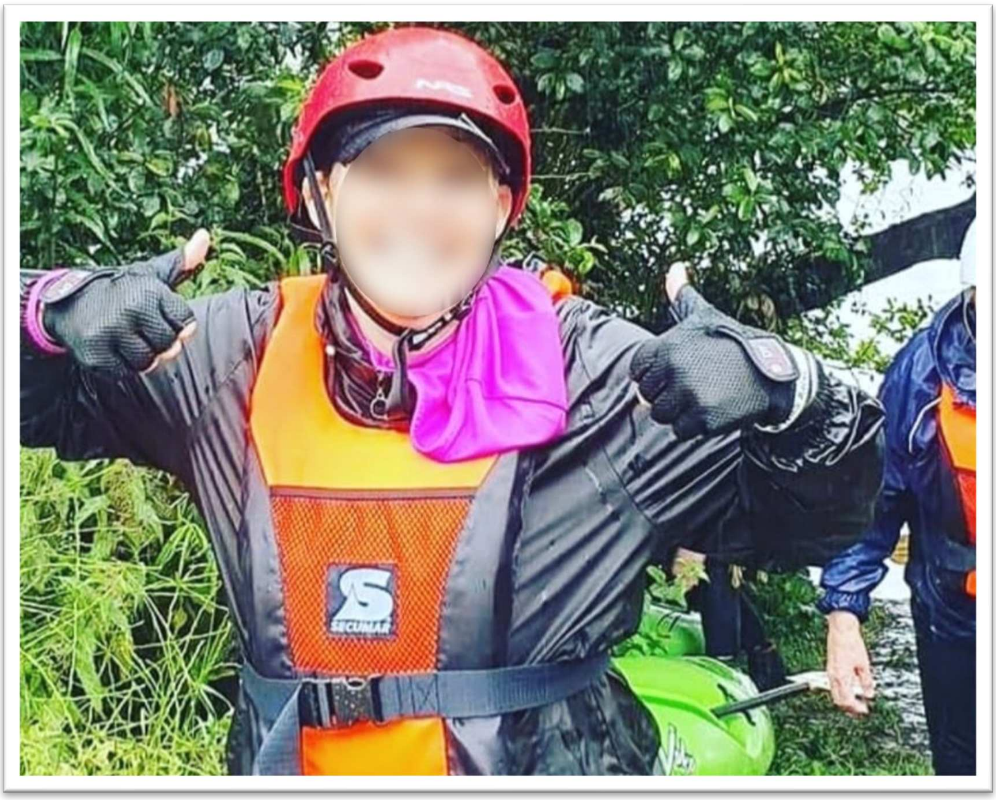


*Figure 35 - Exercise - Food for the body, food for the soul.*

The meaning of nutrition for Irish cancer survivors: A photo voice study

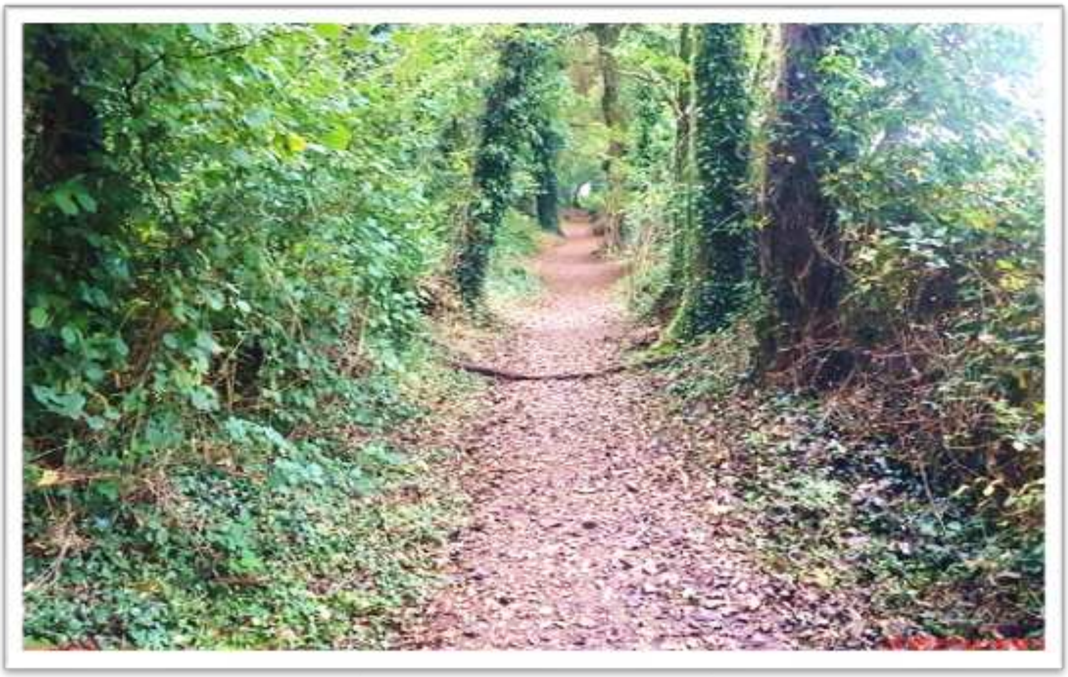

Figure 36 - *This here is de-stress, but it's more, as you see, the very middle centre, that's the light at the end of the tunnel in a cancer journey and the broken branch across the photo signifies the barriers you may encounter, but there is always light at the end of the tunnel*.

The meaning of nutrition for Irish cancer survivors: A photo voice study

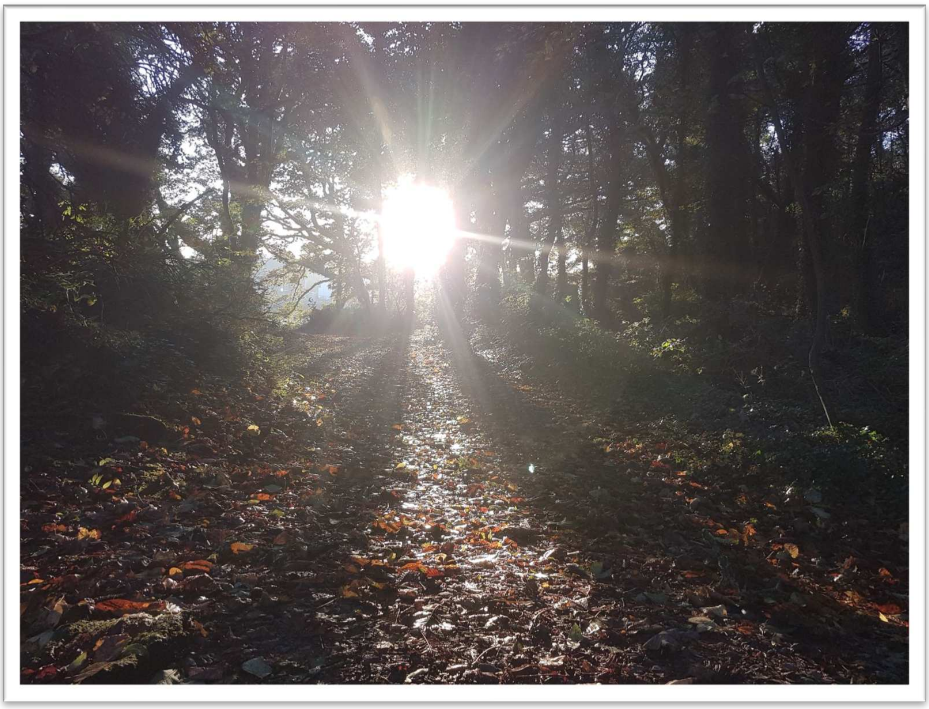

*Figure 37 - Hope*

The meaning of nutrition for Irish cancer survivors: A photo voice study

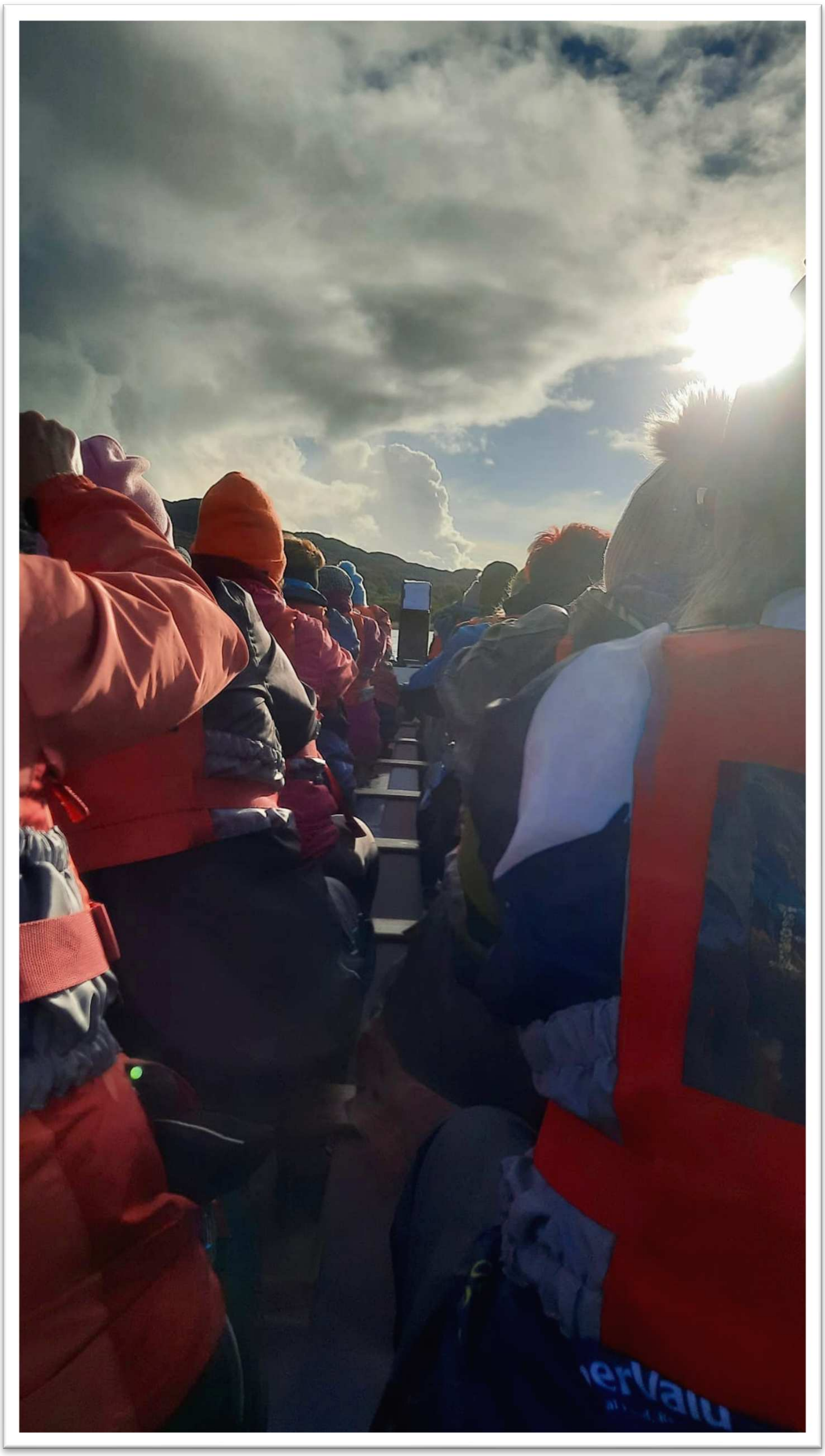

*Figure 38- Working up an appetite*

The meaning of nutrition for Irish cancer survivors: A photo voice study

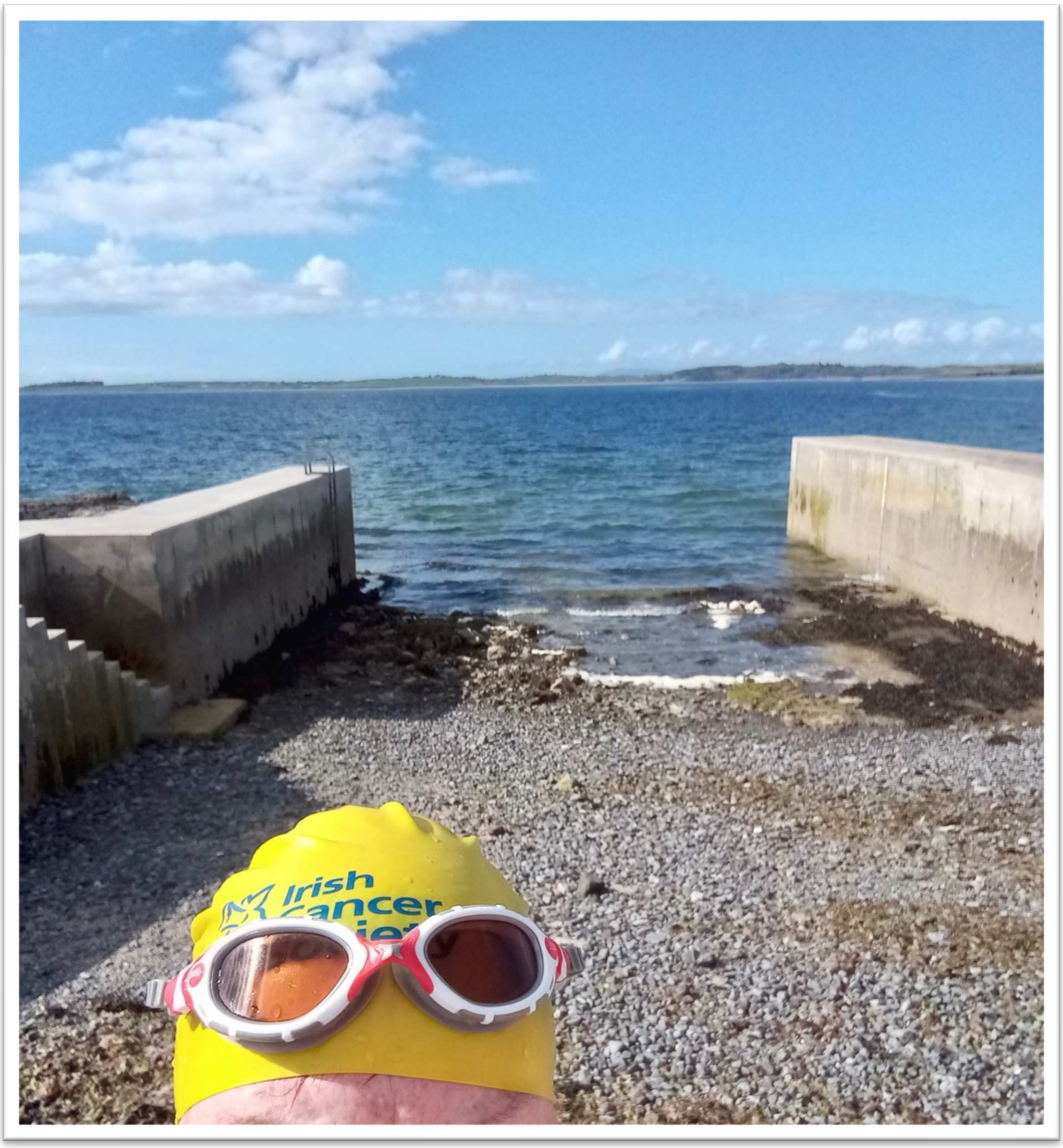


*Figure 39 - The sea is good sauce.*

The meaning of nutrition for Irish cancer survivors: A photo voice study

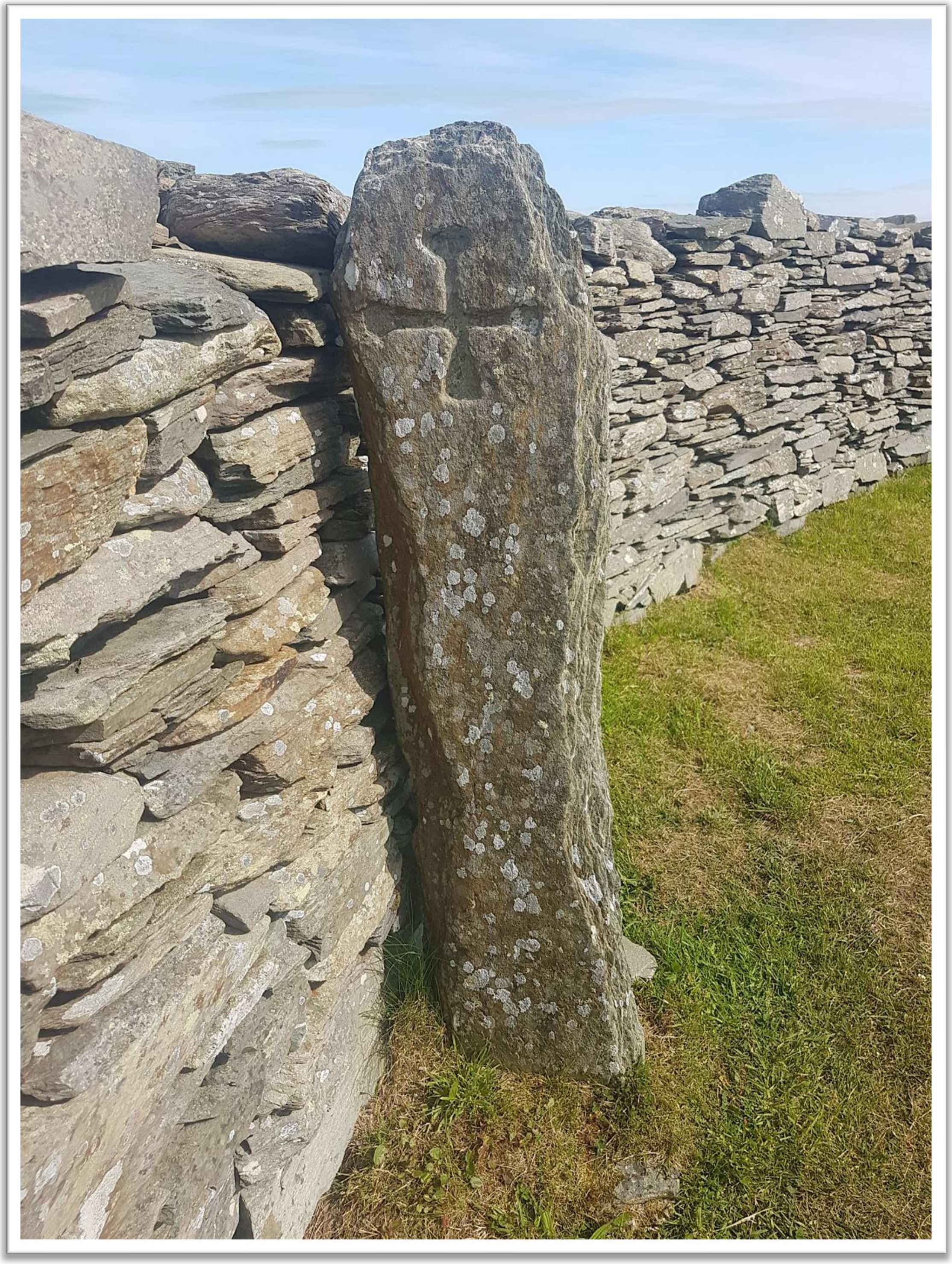

*Figure 40 – Meditation*

The meaning of nutrition for Irish cancer survivors: A photo voice study

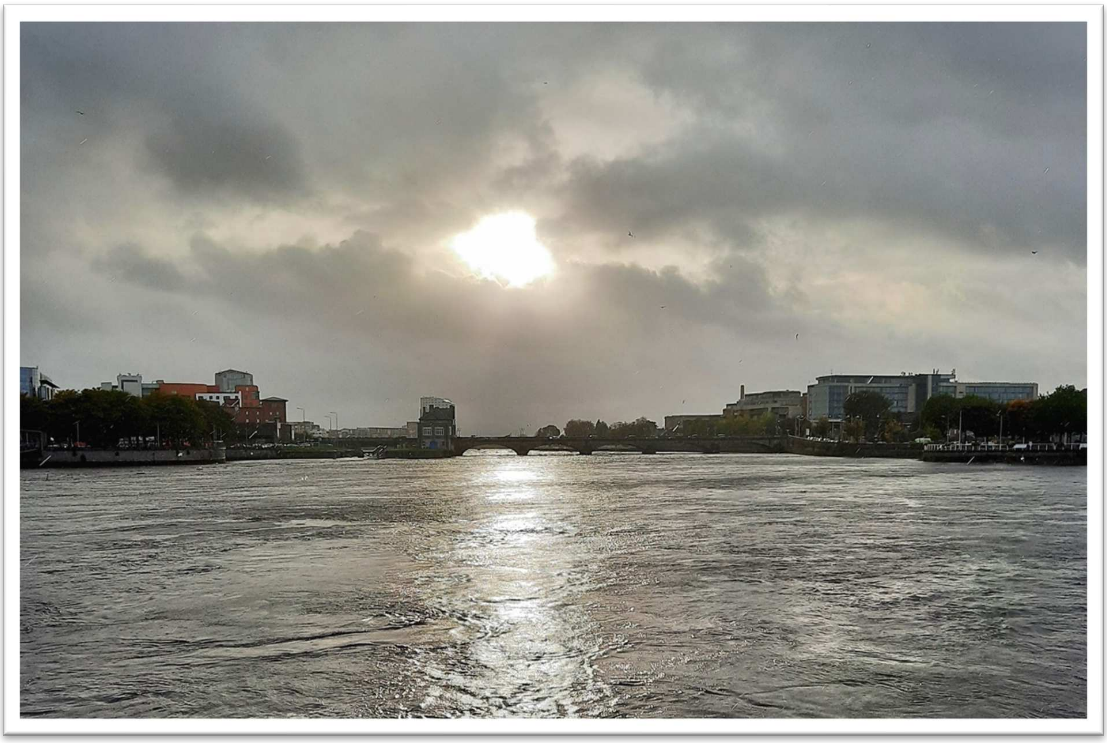

*Figure 41 - Food for the soul.*
